# Molecular and Cellular Changes in the Lumbar Spinal Cord following Thoracic Injury: Regulation by Treadmill Locomotor Training

**DOI:** 10.1371/journal.pone.0088215

**Published:** 2014-02-10

**Authors:** Hae Young Shin, Hyosil Kim, Min Jung Kwon, Dong Hoon Hwang, KiYoung Lee, Byung Gon Kim

**Affiliations:** 1 Department of Brain Science, Ajou University School of Medicine, Suwon, Republic of Korea; 2 Neuroscience Graduate Program, Department of Biomedical Sciences, Ajou University School of Medicine, Suwon, Republic of Korea; 3 Department of Biomedical Informatics, Ajou University School of Medicine, Suwon, Republic of Korea; 4 Department of Neurology, Ajou University School of Medicine, Suwon, Republic of Korea

## Abstract

Traumatic spinal cord injury (SCI) often leads to debilitating loss of locomotor function. Neuroplasticity of spinal circuitry underlies some functional recovery and therefore represents a therapeutic target to improve locomotor function following SCI. However, the cellular and molecular mechanisms mediating neuroplasticity below the lesion level are not fully understood. The present study performed a gene expression profiling in the rat lumbar spinal cord at 1 and 3 weeks after contusive SCI at T9. Another group of rats received treadmill locomotor training (TMT) until 3 weeks, and gene expression profiles were compared between animals with and without TMT. Microarray analysis showed that many inflammation-related genes were robustly upregulated in the lumbar spinal cord at both 1 and 3 weeks after thoracic injury. Notably, several components involved in an early complement activation pathway were concurrently upregulated. In line with the microarray finding, the number of microglia substantially increased not only in the white matter but also in the gray matter. C3 and complement receptor 3 were intensely expressed in the ventral horn after injury. Furthermore, synaptic puncta near ventral motor neurons were frequently colocalized with microglia after injury, implicating complement activation and microglial cells in synaptic remodeling in the lumbar locomotor circuitry after SCI. Interestingly, TMT did not influence the injury-induced upregulation of inflammation-related genes. Instead, TMT restored pre-injury expression patterns of several genes that were downregulated by injury. Notably, TMT increased the expression of genes involved in neuroplasticity (Arc, Nrcam) and angiogenesis (Adam8, Tie1), suggesting that TMT may improve locomotor function in part by promoting neurovascular remodeling in the lumbar motor circuitry.

## Introduction

Patients with moderate to severe traumatic spinal cord injury (SCI) almost invariably suffer from life-long deficits in locomotion. This locomotor dysfunction can be attributed to severance of connections between motor centers in the brain and the lumbar spinal cord. Currently, there is no pharmacological or biological therapeutic option clinically proven to improve locomotor function. The lack of effective treatments in part reflects overwhelming obstacles to promotion of axonal connection between the two motor centers.

An alternative approach is to train intrinsic spinal locomotor circuitry generating rhythmic spontaneous hindlimb movements [1]–[3]. Treadmill locomotor training (TMT) is employed to train and activate locomotor circuits located in the lumbar spinal cord by providing sensory afferent stimulation [4], [5]. First established in spinalized cats [6], [7], the effectiveness of TMT has been demonstrated in rodent models with variable injury severities and training regimens [8]. A recent study showed that epidural stimulation can effectively reactivate the lumbar motor circuitry to become more sensitive to sensory inputs provided by treadmill training (TMT) [9], raising a hope that TMT in combination with electrical stimulation and/or pharmacological neuromodulation [10] could be an effective therapeutic option to improve locomotor function after SCI [11].

Despite documented effects of TMT in animal models, the molecular and cellular mechanisms mediating “below-level” locomotor recovery are not fully understood. Several studies have suggested that elevated neurotrophin levels in the lumbar spinal cord mediate exercise-induced neuroplasticity [12]. However, there is no study that has systematically investigated molecular changes regulated by TMT. Gene expression profiling by microarray has been used in rodent SCI models to systematically reveal cellular and molecular processes leading to spinal cord degeneration and repair [13]–[15]. The current study employed the microarray strategy to examine gene expression changes in the lumbar motor regions following thoracic contusive SCI at the time points when spontaneous locomotor recovery is observed. Moreover, we compared gene expression patterns in the lumbar spinal cord regions between animals with and without TMT to search for molecular factors mediating TMT-induced locomotor recovery.

## Materials and Methods

### Animals and study design

A total of 81 Adult female Sprague Dawley rats (250∼300 g, aged from 9 to 12 weeks old) were used in this study. All animal protocols were approved by the Institutional Animal Care and Use Committee of Ajou University School of Medicine. As a preliminary study to determine time points for microarray study, 8 injured animals each with or without TMT underwent behavioral examination (see below for detailed methods). For microarray analysis, animals were randomly assigned to four groups: 1) animals subjected to laminectomy alone (sham operation) (N = 3), 2) animals subjected to contusive injury and sacrificed 1 week after injury (N = 4), 3) animals subjected to contusive injury and sacrificed 3 weeks after injury (N = 4), 4) animals subjected to contusive injury and TMT beginning at 1 week and lasting until 3 weeks after injury (TMT of 2-week duration) (N = 4). To identify genes regulated by TMT independently of SCI, an additional group of animals (N = 3) was subjected to the sham operation and TMT of 2 week duration beginning at 1 week after the sham operation. Separate sets of animals were generated for verification experiments using RT-PCR and western blots (N = 4 for each group). Five animals for sham group, animals 3 weeks after injury with or without TMT were also generated for immunohistochemical studies. The number of animals in different experiments and the survival time points are provided in detail in Table 1.

**Table 1:** Number of animals in each group and at different time points for various experiments.

Experiment	Group	Survival		
		1 week	3 week	8 week
Behavior assessment	SCI			8
	SCI with TMT			8
Microarray	Sham	3		
	Sham + TMT		3	
	1 week injury	4		
	3 weeks injury		4	
	3 weeks injury + TMT		4	
Quantitative RT-PCR	Sham	4		
	1 week injury	4		
	3 weeks injury		4	
	3 weeks injury + TMT		4	
Western blot	Sham	4		
	1 week injury	4		
	3 weeks injury		4	
	3 weeks injury + TMT		4	
Histological assessment	Sham	5		
	3 weeks injury		5	
	3 weeks injury + TMT		5	

SCI: spinal cord injury, TMT: treadmill training.

### Surgical procedures, TMT protocol, and behavioral assessment

To create contusive spinal cord injury model, animals were anesthetized with chloral hydrate (400 mg/kg, i.p.) during all surgical procedures. A dorsal laminectomy was performed at the ninth thoracic vertebral level (T9) to expose the spinal cord. Then animals were subjected to contusion injury by mechanical impact with a force of 200 kdyn using an Infinite Horizon Impactor (Precision System and Instrumentation, Lexington, KY, USA). Prophylactic antibiotics were administered by intraperitoneal injection on the day after each surgery, and bladder care was provided twice daily until spontaneous voiding resumed. TMT was performed in the Flat Treadmill System for rats with 4 channels (Model, IW-FT; IWOO Scientific Corporation, Seoul, Korea). Each channel consists of a 14 cm by 45 cm runway with an electrical stimulator installed at the end of the runway. Electric shocks of 1.2 mA were applied when an animal was not walking and displaced to the end of its runway. Typically, electric shocks were required several times on the first day of training, but were rarely applied after a few days. Although animals could not perform fully weight-supported plantar stepping when TMT was initiated at 1 week after injury, they were able to walk without auxiliary support at a slow speed (5 meters per minute). As locomotion was improved, the belt speed was gradually increased to 12 meters per minute during the first week and maintained at this level until the end of training. TMT was performed daily for 14 days with each session lasting 30 minutes. Locomotor recovery was assessed using the Basso, Beattie, and Bresnahan (BBB) locomotor rating scale. BBB test was carried out by observers blinded to the treatment conditions. BBB test was performed on each animal on the day following contusion and once a week thereafter until 8 weeks after.

### RNA isolation for microarray

On the day of sacrifice, animals were anesthetized with an overdose of chloral hydrate and briefly perfused with ice-cold saline to remove blood components from the tissue. Spinal cord tissue containing the epicenter and the entire length below was quickly dissected. The dissected spinal cord was cut at the beginning of the lumbar enlargement, and the lumbar spinal cord block extending 1 cm distal to the initial cut was collected. This tissue block contained the rostral portion of the sacral segments but not the conus medullaris. Care was taken to remove the stubs of the lumbosacral roots attached to the cord. Dissected lumbar spinal cord blocks were frozen rapidly and stored at –70°C until use. Total RNA was isolated using an RNeasy Microarray Tissue Mini kit (Qiagen, Hilgen, Germany) in accordance with the manufacturer's instructions. The concentration of the RNA samples was quantified using a Nanodrop 1000 (Thermo Fisher Scientific, Wilmington, DE, USA). RNA quality was assessed using a Bioanalyzer 2100 (Agilent technologies Inc., Santa Clara, CA, USA), and RNA samples with RNA integrity number (RIN) higher than 7.5 were judged as acceptable. All of the samples prepared in our study had RIN value higher than 8.

### Microarray analysis

For each sample, 6 µg of RNA were used as input into the Affymetrix procedure as recommended by the manufacturer’s protocol (http://www.affymetrix.com). Briefly, 6 µg of total RNA was converted to double-strand cDNA using oligo (dT) primer incorporating a T7 promoter. Amplified RNA (cRNA) was generated from the double-stranded cDNA template though an IVT (in-vitro transcription) reaction and purified with the Affymetrix sample cleanup module. After fragmentation, RNA was hybridized to the arrays containing over 22,500 probe sets. After hybridization, the chips were stained and washed in a Genechip Fluidics Station 450 and the microarray signals were scanned by using a Genechip Array scanner 3000 7G. The robust multi-array average approach with a quantile normalization algorithm was applied to extract probe-level expression intensities of genes.

### Data analysis

For gene-level expression, we calculated the mean value of the probe-level intensities for each gene. Differentially expressed genes were identified when individual genes met the following two conditions: 1) there was at least a 1.5-fold change in expression level of the genes between groups and 2) there was a statistically significant difference in the expression level using Student's t-test (*p*<0.05). Pathway and Gene Ontology-based functional enrichment analysis using DAVID (the Database for Annotation, Visualization and Integrated Discovery) [16] was employed by using differentially expressed genes from each pair-wise comparison among sham, injury, and TMT groups (cut-off threshold of significance: *p*<0.1). For clustering analysis, the k-means clustering algorithm implemented in the MEV (Multi Experiment View) software package [17] was applied to the genes that were differentially expressed between the 3 week post-injury and TMT groups. To identify and visualize the injury-related protein network modules, we used Cytoscape software version 2.8.3 [18]. For the rat protein-protein interactions we compiled diverse databases including BIND, BIND_t, BioGRID, CORUM, DIP, HPRD, IntAct, MINT, MPPI, OPHID, InnateDB, and MatrixDB. We also included human protein-protein interactions after converting human proteins to rat proteins using human-rat orthologs of the InParanoid database [19]. Our microarray data have been deposited in the NIH Gene Expression Omnibus database with accession code GSE52763.

### Quantitative reverse transcription polymerase chain reaction (RT-PCR)

Expression changes of genes of interest were verified by quantitative real-time RT-PCR. Total RNA was extracted from lumbar spinal cords using Trizol (Qiagen, Hilgen, Germany) according to the manufacturer’s protocol. Oneµg of RNA was reverse transcribed to cDNA using a standard reverse transcription protocol. Oneµl of cDNA was added to SYBR Green Master Mix (Dakara, Otsu, Shiga, Japan) containing primer pairs at 10 pM. Primer sequences are listed in Table 2. Quantitative real-time PCR was performed according to the manufacturer’s protocol using a 7500 Real-Time PCR System (Applied Biosystems, Foster City, CA, USA). Cycling conditions were 94°C for 30s, 53°C ∼ 64°C for 31s, and 72°C for 60s with a total of 34 cycles. Melting curves were generated after the last extension step, and Ct values were calculated by the Applied Biosystems 7500 software. Ct values of target genes were normalized to the housekeeping gene 18Sr and analyzed using the 2^−▵▵Ct^ method [20]. Using the 2^−▵▵Ct^ method, the data are presented as the fold change in gene expression normalized to an endogenous reference gene and relative to the control.

**Table 2:** Primer sequences used for real time RT-PCR.

Gene	5' primer (Forward)	3' primer (Reverse)
18S rRNA	CGGCTACCACATCCAAGGAA	TGCTGGCACCAGACTTGCCCTC
Galectin3	AGCCCAACGCAAACAGTATC	GGCTTCAACCAGGACCTGTA
C3	CAGCAGACCTCAGTGACCAA	GCCTCTTCTCTAGGCCGAAT
C1qa	GGCCTAGAAGCATCATAGAACACGA	CACTCCACTGTGTCTTCATCAGCTC
C1qb	GGCCTAGAAGCATCATAGAACACGA	CACTCCACTGTGTCTTCATCAGCTC
Timp1	ACAGGTTTCCGGTTCGCCGCC	CTGCAGGCAGTGATGTGCAA
Itgb2	AAGGGAGTCATGGAGTGTGG	TCACTTTGTTGGGGATGTCA
March6	AGGGACCACAATGTTTCTGC	GGAAGCCAGAGCATCAGAAG
Kdm4c	GCCCATGACTGTGAAGGAGT	CGCACTCTTCTTCAACCACA
Frg1	TCCAGTCCTCCAGAGCAGTT	GCCATCTTCCCATCTTGAAA
Adam8	GCAGCCAGGTTGACCTAGAG	ATTCATGACAACAGGCACCA
Nrcam	GGCAGCAAAGAAGAATGGAG	CTTGGGTCGCAATATCCACT
Arc	CTGAGATGCTGGAGCACGTA	GCCTTGATGGACTTCTTCCA
Tie1	AAGGTCACACACACGGTGAA	CATTTTGGAGCTGCAGTTGA

### Tissue processing and immunohistochemistry

Rats were anesthetized with an overdose of chloral hydrate and perfused with phosphate buffered saline (PBS) followed by 4% paraformaldehyde (PFA) in 0.2 M phosphate buffer. The spinal cord tissues was separated and post-fixed in 4% PFA for 2 hours, followed by cryoprotection in a graded series of sucrose solutions. For lumbar spinal cord tissue, 20 µm thick transverse cryosections were made in a 1∶10 series. Tissue sections were mounted onto Superfrost plus slides (Fisher Scientific, Pittsburgh, PA, USA), and stored at –20°C until use. For immunohistochemistry, tissue sections were treated with 10% normal goat serum and 0.3% triton X-100 for one hour, and then primary antibodies dissolved in the same blocking solution were applied at 4°C overnight. The primary antibodies were rabbit anti-Iba1 (1∶500; Wako, LA, CA, USA), mouse anti-synaptotagmin (1∶500; Chemicon, Temecula, CA, USA), rabbit anti-C3 (1∶100; MP Biomedicals, Aurora, OH, USA), mouse anti-CD11b (1:500; Serotec, Oxford, UK), and mouse anti-MAP2 (1∶500; Sigma, St Louis, MO, USA). Tissue sections were washed thoroughly and then incubated with appropriate secondary antibodies tagged with Alexa Fluor 488 or 594 (1∶500; Molecular Probes, Eugene, OR, USA) for one hour at room temperature. Coverslips were mounted onto the slides with glycerol-based mounting medium (Biomeda, Foster City, CA, USA). Images were taken using a FV 300 confocal microscope (Olympus, Tokyo, Japan). For brightfield imaging of Iba1 immunostaining, transverse spinal cord sections were treated with 10% hydrogen peroxide to quench endogenous peroxidase activity. After rinsing and blocking, slides were incubated with rabbit anti-Iba1 (1∶500; Wako, LA, CA, USA) at 4°C overnight in a humid chamber. Tissue sections were rinsed and incubated with biotinylated goat anti-rabbit IgG secondary antibody (1∶400), and the antigen-antibody reaction was visualized using a Vectastain Elite ABC kit (Vector, Burlingame, CA) with a Vector SG peroxidase substrate kit (Vector, Burlingame, CA). Eriochrome cyanine staining was used to define the boundary of gray and white matter.

### Western blot

Lumbar spinal cord tissue blocks were dissected using the same methods as for the microarray experiment. The tissue blocks were homogenized in ice-cold lysis buffer containing 20 mM Tris-HCl (pH 7.5), 1 mM EDTA, 5 mM MgCl_2_, 1 mM dithiothreitol, 0.1 mM phenylmethylsulfonyl fluoride, and a protease inhibitor cocktail (Pierce, Rockford, IL, USA). The tissue homogenate was centrifuged at 10,000 rpm for 15 min at 4°C, and the protein concentration of the supernatant was measured using a Bradford assay. Equal amounts of proteins were resolved by SDS-PAGE and transferred to a PVDF membrane (Millipore, Bedford, MA). The membrane was blocked in Tris-buffered saline (TBS) containing 5% BSA and probed with rabbit anti-Arc (1:500; Santa Cruz, Dallas, USA) and rabbit anti-Adam8 (1∶500; Millipore, Bedford, MA, USA). After washing, the membranes were incubated for 2 hrs at room temperature with secondary antibodies (goat anti-rabbit, 1∶3000; Cell Signaling, Danvers, MA, USA). All blots were probed with antibodies against β-actin (1∶20000; Sigma, St Louis, MO, USA) to normalize differences in loading amount.

### Quantitative image analysis

For unbiased stereological evaluation of the number of microglia in the lumbar spinal cord sections, Iba1-positive cells were counted using an Olympus BX51 Microscope coupled with Stereo Investigator 8 software (MBF Bioscience, Williston, VT, USA). Cell counting was performed using one out of 10 serial slides (inter-section interval in one slide was 200 µm). In one slide, every 4th section was used for counting (section sampling fraction, ssf  = 1/40). Iba1- positive cells within optical dissectors placed randomly in regions of interest were counted using stereological criteria. Lumbar spinal cord was delineated using a 4x objective, and optical dissectors consisting of an inclusion and an exclusion line were randomly placed. Based on the eriochrome myelin staining, white matter and gray matter were delineated separately. The sampling grid dimensions were 200×200 µm^2^ and the counting grid was 120×120 µm^2^, resulting in an area sampling fraction (asf) of 0.36. Using a dissector height of 12 µm, height sampling fraction (hsf) was 0.67 (12/18) with the average postprocessing section thickness 18 µm. Actual counting was performed using 40x magnification. A total of 5 animals from each group were used for quantification. To quantify the proportion of microglia and synaptotagmin co-localization, a total of three sections were chosen per each animal (5 animals per group). Five regions of interest were captured from the same transverse sections at 63x magnification using an LSM 510 confocal laser-scanning microscope. Counting of synaptotagmin puncta colocalizing with Iba1 immunoreactivity was performed in a single optical plane. The total number of colocalized synaptotagmin puncta was divided by the microglia number obtained in all 15 images (5 images per section, 3 sections).

### Statistical methods

Statistical analysis was performed using GraphPad Prism software version 5.0 (San Diego, CA). Unpaired Student's t-tests or one-way ANOVA followed by Tukey's post-hoc test was used to compare group means at single time points. Repeated measures two-way ANOVA was used to compare differences in BBB locomotor scores over different time points. Numerical values in all the graphs are presented as mean ± SEM.

## Results

### TMT improved locomotor recovery after thoracic SCI

**Figure 1:**
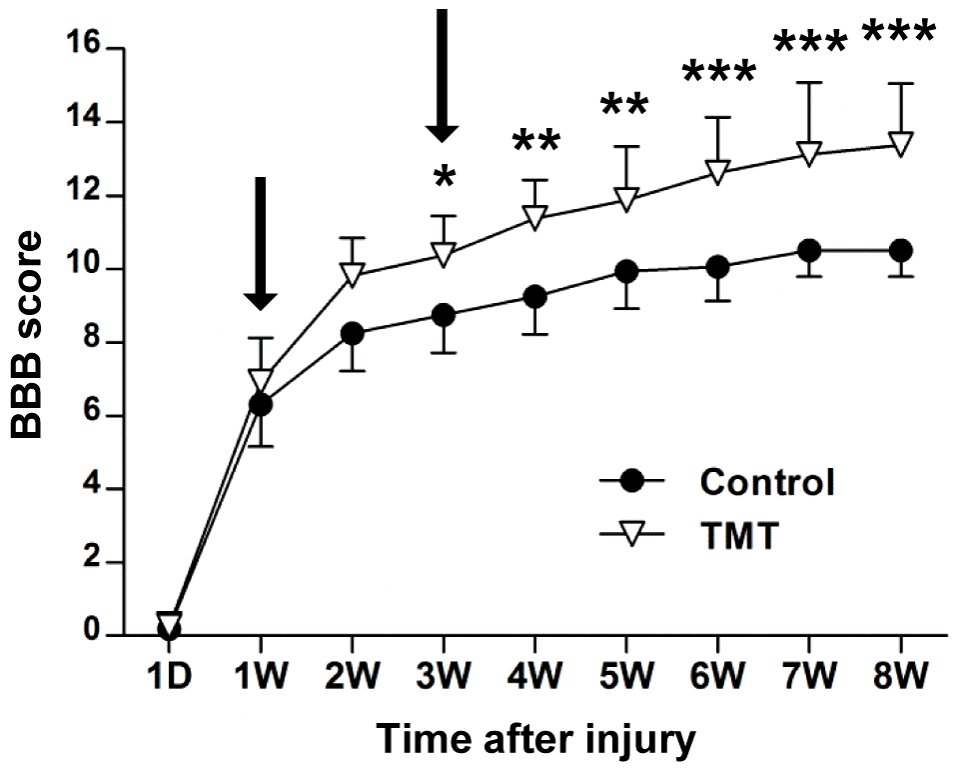
Treadmill training promotes locomotor recovery following contusive injury. Locomotor recovery was assessed by Basso, Beattie, and Bresnahan (BBB) locomotor scoring over the 8-week period after thoracic contusive injury. Animals subjected to treadmill training (TMT) showed enhanced locomotor recovery compared to those without TMT (control group). Arrows indicate the two time points chosen for microarray gene analysis. *, **, and *** indicate *p*<0.05, *p*<0.01, and *p*<0.001, respectively, by Bonferroni post-hoc tests following repeated measures two-way ANOVA. N = 8 animals per group.

We first documented positive effects of our treadmill training (TMT) protocol on locomotor recovery after contusive thoracic SCI. Animals showed rapid locomotor recovery during the first week regardless of TMT (Figure. 1). Recovery in control animals (without TMT) measured using the BBB locomotor score was rapid until around 3 weeks, after which only minimal improvement was observed. Animals with TMT exhibited enhanced locomotor recovery beginning at 2 weeks and continued to show higher quality overground locomotion (Figure. 1). The locomotor improvement induced by TMT was statistically significant compared to control animals (*p*<0.001 by repeated measures two-way ANOVA). To gain an insight on the molecular mechanisms at the spinal level underlying the locomotor recovery, we performed gene expression profiling in the lumbar motor region where the spinal locomotor center is located. Seventh day after injury was chosen as an early time point because animals typically showed rapid locomotor improvement around this time. We also chose 21st day as a late time point when spontaneous locomotor recovery began to plateau and the effects of our TMT protocol became evident (Figure. 1). We first analyzed gene expression patterns in the lumbar motor region following thoracic SCI (T9 level) at these two time points.

### Gene expression profiling in the lumbar spinal cord below the lesion

**Figure 2:**
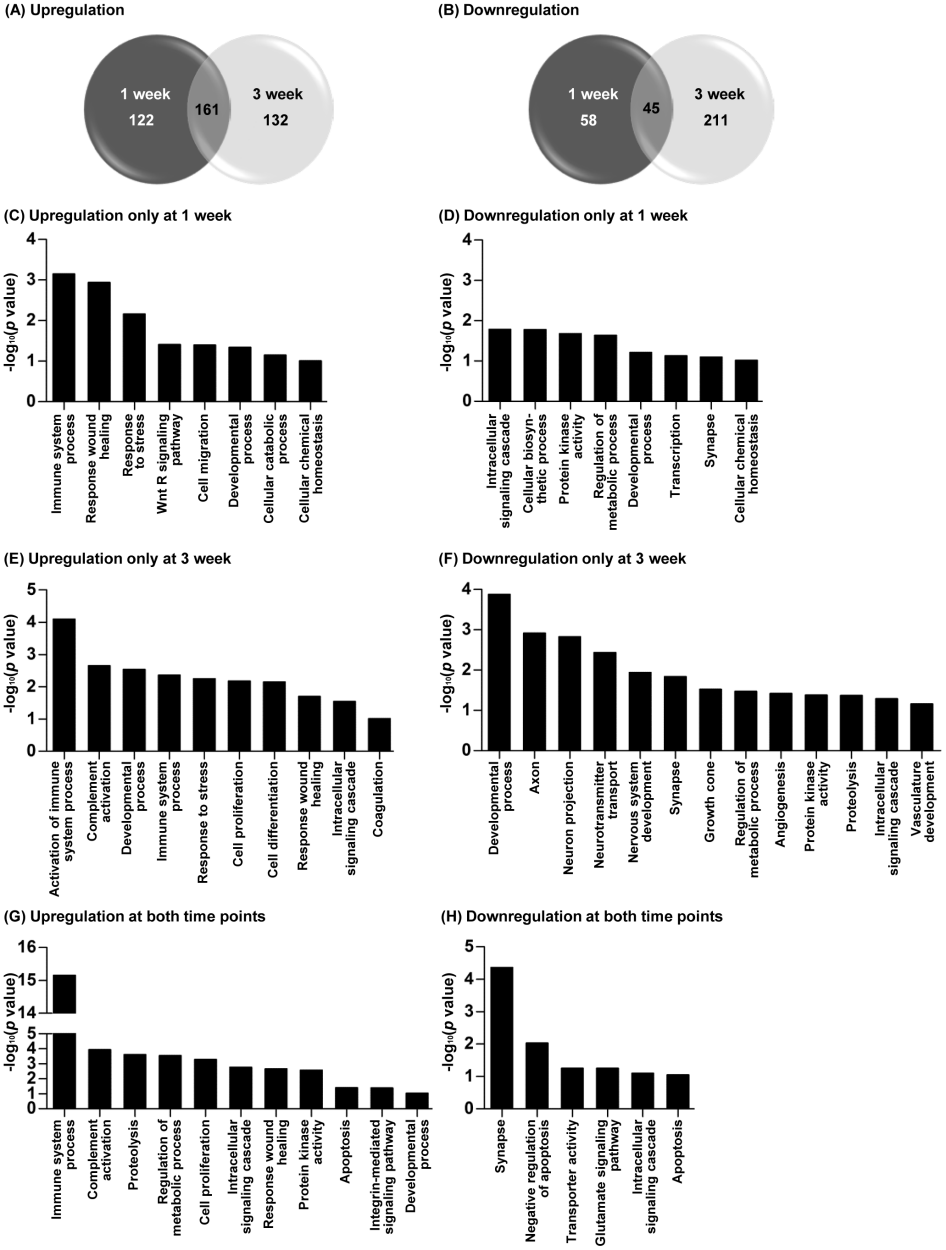
Gene expression profile changes in the lumbar spinal cord at 1 and 3 weeks after thoracic spinal cord injury. (A-B) The number of genes differentially expressed (A, upregulated or B, downregulated) at 1 or 3 weeks after injury compared to sham operated animals. Venn diagrams show the total number of differentially expressed genes at 1 (dark gray circle) and 3 weeks (light gray circle) after injury. The number in the area of overlap of the two circles indicates the number of genes whose expression changed at both time points. (C-H) Gene ontology (GO)-based functional enrichment analysis using DAVID software. GO terms significantly (*p*<0.1) enriched in the following groups of genes are shown on the X-axis; upregulation only at 1 week (C), downregulation only at 1 week (D), upregulation only at 3 week (E), downregulation only at 3 week (F), upregulation at both time points (G), downregulation at both time points (H). Log_10_- transformed *p* values are shown on the Y-axis.

Contusive SCI led to an upregulation of almost 300 genes in the lumbar motor region at either 1 or 3 weeks after injury, 161 of which were upregulated at both time points (Figure. 2A). The number of downregulated genes at 1 week was smaller than that for upregulation at the same time point (Figure. 2B). However, the number of downregulated genes at 3 weeks was comparable to that for upregulated genes at the same time point. Intriguingly, the number of genes downregulated only at the 3- week time point was larger than that of genes downregulated only at 1 week, suggesting that more widespread transcriptional depression occurred by the later time point. Complete lists of genes that were differentially expressed in the lumbar motor regions after thoracic SCI are provided in Tables S1-3. Gene ontology analysis revealed that genes related to development, neuronal elements (such as axon, neuronal projection, and growth cone), neurotransmitter transport, metabolic process, and angiogenesis were preferentially downregulated at the 3-week time point (Figure. 2F). At the 1-week time point, genes involved in metabolic functions (cellular biosynthetic process, regulation of metabolic process), developmental process, and synapse were downregulated (Figure. 2D). Notably, genes related to synaptic function, such as Grin1 (encoding NMDA receptor subunit 1), Arc, Grm7 (encoding metabotropic glutamate receptor 7), and Syt6 (encoding synaptotagmin-6), were significantly downregulated at both 1 and 3 weeks (Figure. 2H).

The most striking finding in the expression profiling was the upregulation of genes with functions in immune processes and/or inflammatory reactions (Figure. 2C, 2E, and 2G). Biological processes related to this category include regulation of immune responses, immune cell (B cell, T cell, and leukocytes) proliferation and differentiation, cytokine production, the complement system, and inflammatory signaling pathways. Some of the genes related to inflammatory processes were upregulated at either 1 or 3 weeks after injury, but the majority of these genes were upregulated at both time points. We focused our analysis on the inflammation-related genes that showed robust (more than 2-fold) upregulation at both time points (Table 3). Galectin3 (Lgals3), which is selectively expressed in microglial cells after SCI and thought to contribute to secondary injury [15], [21], showed the most robust upregulation at both time points. Genes encoding markers for immune cells such as CD68, CD86, CD45 (Ptprc), and CD53 [22]–[24], were also upregulated. Matrix metalloproteinases (MMPs) regulate post-injury inflammation in the spinal cord contributing to diverse pathological outcomes [25]. We found that the Timp1 gene, an endogenous tissue inhibitor of metalloproteinases, was potently upregulated.

**Table 3:** Inflammation-related genes that were upregulated by more than 2-fold at both 1 and 3 weeks after SCI.

Gene symbol	Gene full name	Log2 (fold change)		
		1 week	3 week	TMT
Lgals3	lectin, galactoside-binding, soluble, 3	2.53	3.41	3.34
C3	complement component 3	2.25	1.64	1.62
Cd68	Cd68 molecule	2.20	2.61	2.58
Cd86	Cd86 molecule	2.20	2.61	1.39
Aif1	allograft inflammatory factor 1	2.13	1.73	1.77
Timp1	TIMP metallopeptidase inhibitor 1	2.11	1.87	1.63
Ly86	lymphocyte antigen 86	2.03	1.34	1.44
Cfh	complement factor H	1.74	1.88	1.76
Ptprc	protein tyrosine phosphatase, receptor type, C (CD45)	1.74	1.14	1.30
Blnk	B-cell linker (Ly57)	1.72	1.60	1.72
Tifab	TRAF-interacting protein with forkhead-associated domain, family member B	1.66	1.16	1.35
Csf3r	colony stimulating factor 3 receptor (granulocyte)	1.63	1.30	1.42
Hmox1	heme oxygenase (decycling) 1	1.59	1.06	0.83
Klhl6	kelch-like family member 6	1.53	1.04	1.01
Pycard	PYD and CARD domain containing	1.44	1.11	1.19
C1qb	complement component 1, q subcomponent, B chain	1.44	1.49	1.46
Itgal	integrin, alpha L	1.42	1.14	1.54
Nckap1l	NCK associated protein 1 like (Hem1)	1.42	1.18	1.04
Ptpn6	protein tyrosine phosphatase, non-receptor type 6 (Shp1)	1.38	1.29	1.23
Fcgr2a/2b	Fc fragment of IgG, low affinity lia/b, receptor (CD32)	1.36	1.51	1.76
Cd53	Cd53 molecule	1.34	1.25	1.36
C1qa	complement component 1, q subcomponent, A chain	1.33	1.46	1.37
C2	complement component 2	1.33	1.40	1.16
Inpp5d	inositol polyphosphate-5-phosphatase D (Ship1)	1.26	1.21	1.16
Csf1r	colony stimulting factor 1 receptor	1.26	1.10	1.28
Mall	mal, T-cell differentiation protein-like	1.18	1.37	1.41
Anxa3	annexin A3	1.15	1.00	1.13
Mrc2	mannose receptor, C type 2	1.13	1.36	1.21
Cyba	cytochrome b-245, alpha polypeptide (p22-phox)	1.12	1.11	1.23
C1qc	complement component 1, q subcomponent, C chain	1.10	1.04	1.18
Itgb2	integrin, beta 2	1.03	1.29	1.36
Ifitm1	interferon induced transmembrane protein 1	1.01	1.28	0.80

TMT  =  treadmill locomotor training.

**Figure 3:**
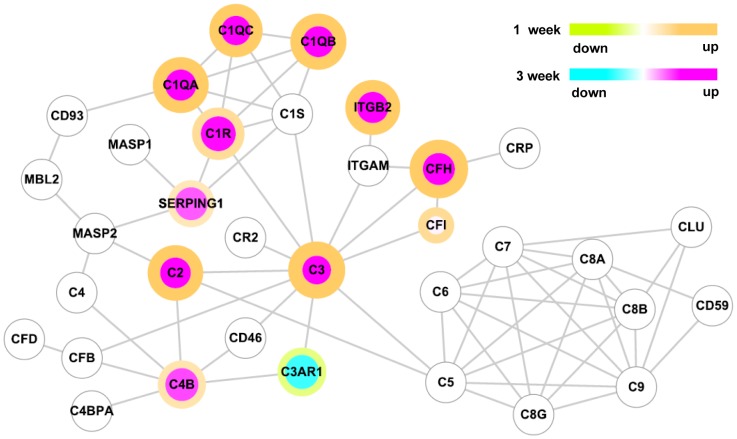
The complement pathway protein-protein interaction network. Nodes represent proteins and lines represent physical interactions between them. The color of the node indicates the fold-change in gene expression at 3 weeks relative to that in the control sham group. The color of the node border indicates the fold-change in expression at 1 week relative to that in the control sham group. Color scales are shown in the upper right. The fold-changes were log_2_-transformed and only genes that showed significantly different expressions (*p*<0.05 by Student’s t-test and absolute fold-change > 1.5) were color-mapped.

Particularly intriguing was the upregulation of several molecules that comprise components of the complement system. Of these, C3 showed the most robust upregulation (more than 4-fold and 3-fold at 1 and 3 weeks, respectively). All three subunits of C1q complexes and C2 were also robustly upregulated. When the changes in gene expression were mapped on a diagram of the protein interaction network of the complement pathway, most of the complement components that participate in a cascade (both classical and alternative) leading to activation of C3 showed increased expression (Figure. 3). In contrast, the complement components that participate in the assembly of membrane attack complex were not significantly changed. C3 activation plays a pivotal role in opsonization of pathogens which are then recognized by complement receptors on macrophages and ultimately phagocytosed [26]. Itgb2 (intergrin beta-2 subunit, CD18), which is a component of complement receptor 3 (CR3) and mediates C3-CR3 interaction in the phagocytic process [26], was also significantly upregulated (Table 3). Expression of the Serping1 (C1 inhibitor) and Complement factor H (Cfh) genes, encoding complement control proteins regulating the classical and alternative pathways [27], respectively, were increased as well (Table 3 and Figure. 3).

**Figure 4:**
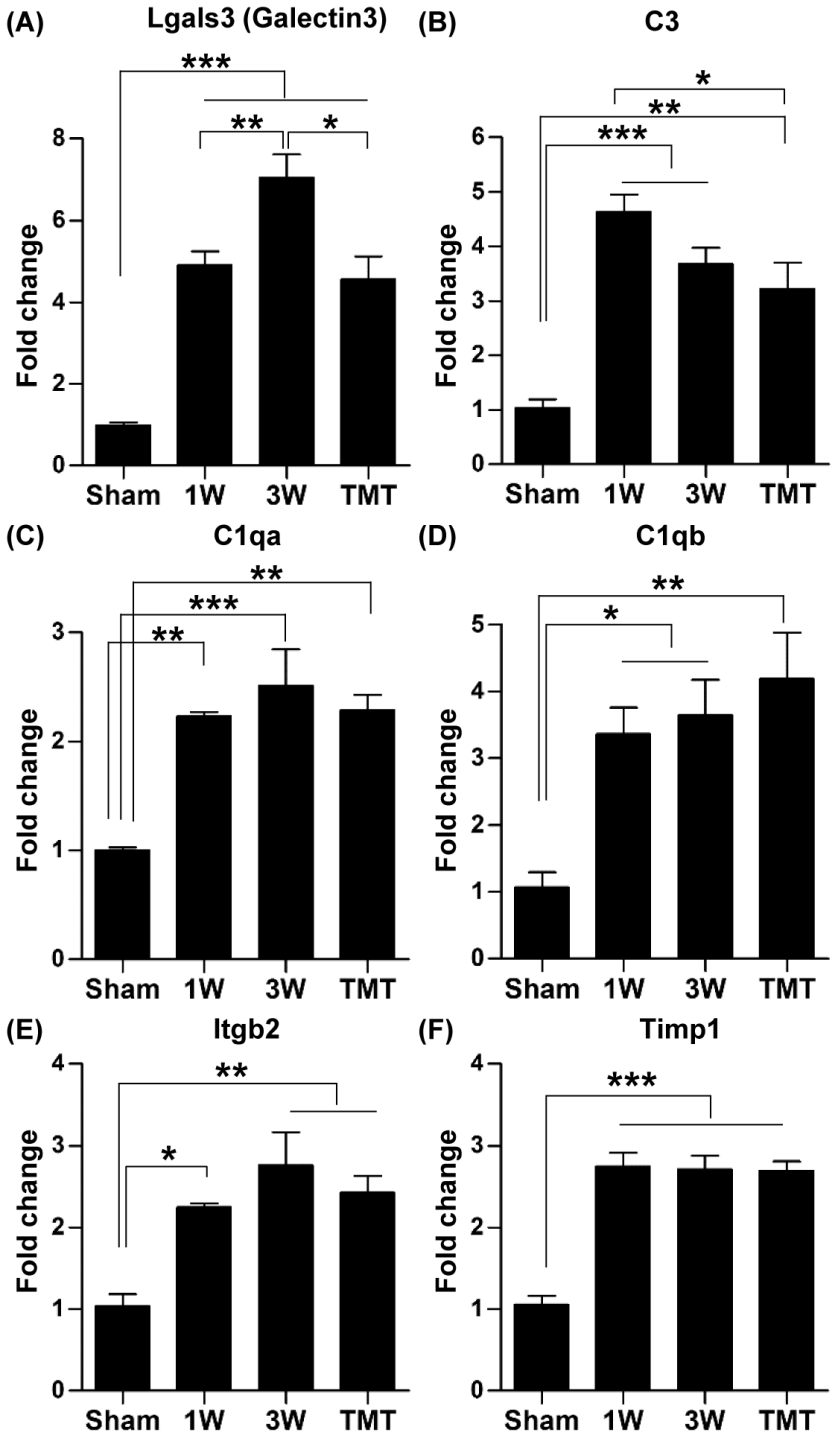
Validation of microarray data for inflammation-related genes using real time RT-PCR. (A-F) Quantification graphs of real-time RT-PCR for Lgals (Galectin3) (A), C3 (Complement component 3) (B), C1qa (complement component 1, q subcomponent, A chain) (C), C1qb (complement component 1, q subcomponent, B chain) (D), Itgb2 (integrin, beta 2, complement receptor 3 subunit) (E), and Timp 1 genes (F) in the sham operated group (sham), animals 1 week (1W), 3 weeks (3W) after injury without treadmill training (TMT), and with TMT. N = 4 animals for each group. *, **, and *** represent *p*<0.05, *p*<0.01, and *p*<0.001, respectively, by one way ANOVA followed by Tukey's post-hoc analysis. Error bars represent SEM.

Robust upregulation of the Galectin3, C3, C1qa, C1qb, Itgb2 and Timp1 genes was verified by real-time RT-PCR (Figure. 4). For these inflammation-related genes upregulated by SCI, we compared expression levels between animals with or without TMT (at the 3-week time point). Microarray data showed that the elevated levels of expression of inflammation-related genes were maintained in the animals subjected to TMT (Table 3). Real-time RT-PCR also showed that the expression of the principal genes related to inflammation was not appreciably affected by TMT, except Galectin3 where a slight, but significant decrease in expression level was observed in animals with TMT (Figure. 4).

### Potential roles of microglial cells in the lumbar spinal cord below the lesion

**Figure 5:**
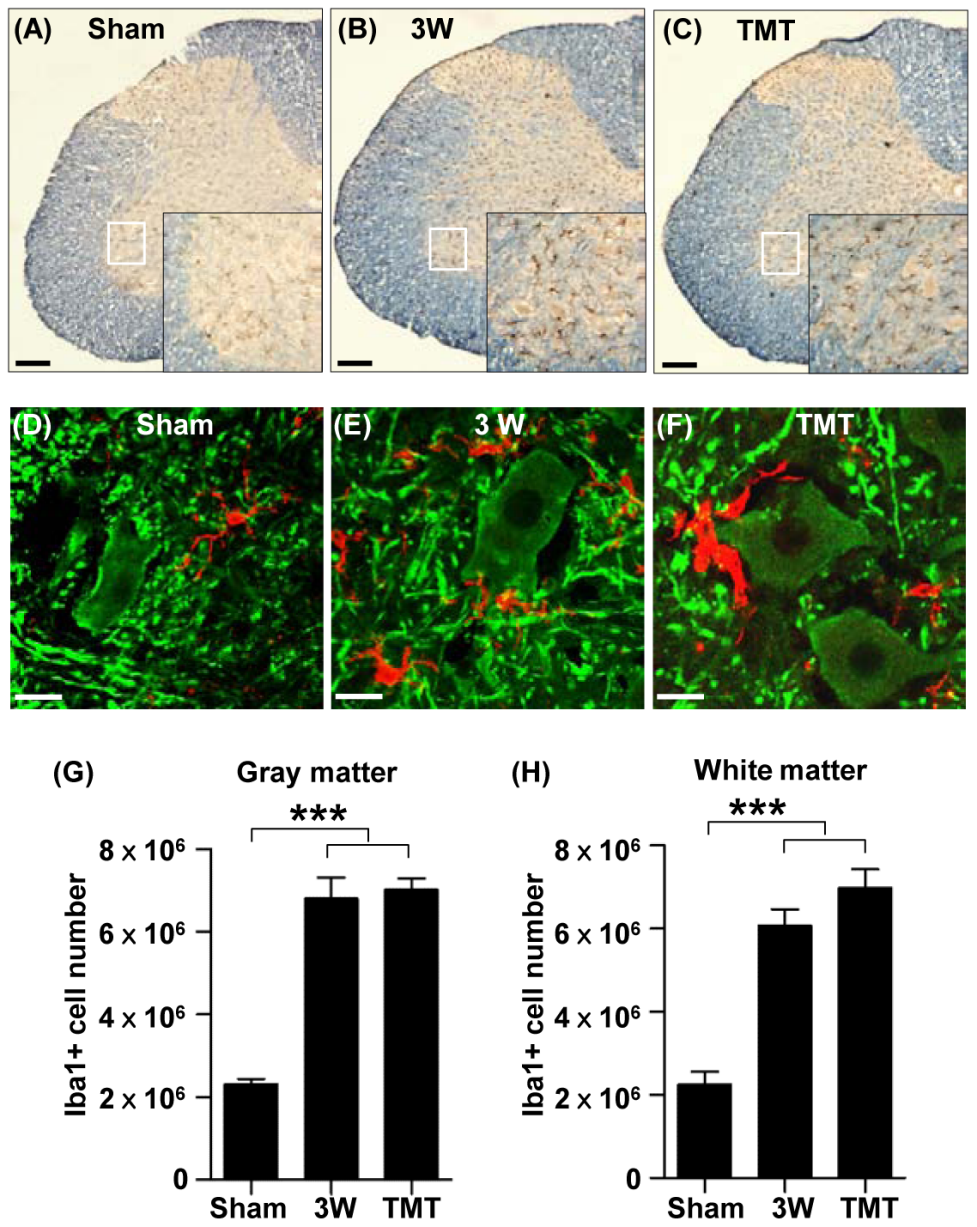
Microglia markedly increased in number in the lumbar motor region following thoracic SCI. (A-C) Representative images of lumbar spinal cord sections stained with an antibody recognizing the microglial marker Iba1 (dark brown) antibody in sham operation (A), at 3 weeks (3W) after injury (B), and at 3W after injury with treadmill training (TMT) (C). Immunostained sections were counterstained with eriochrome cyanine to differentiate the gray matter and white matters. Insets are magnified images of the regions in the white boxes in the ventral gray matter. Scale bars represent 100 µm. (D-F) Representative images of lumbar spinal cord sections colabeled with Iba1 (red) and MAP2 (green) antibodies in sham operation (D), at 3W after injury (E), and at 3W after injury with TMT (F). Note that Iba1 positive microglial cells are frequently associated with MAP2 positive neurons and dendritic neuropil areas. Scale bars represent 20 µm. (G-H) Quantification of stereological counting of Iba1-positive microglial cells in the gray matter (G) and white matter (H) of the lumbar spinal cord. *** represent *p*<0.001 by one-way ANOVA followed by Tukey's post-hoc analysis. N = 5 for each group. Error bars represent SEM.

The increased expression of galectin3 and the myeloid cell marker CD68 suggested that microglia might increase in number in the lumbar motor region following thoracic SCI. Indeed, immunohistochemical staining showed a marked increase in the number of Iba1-positive microglia at 3 weeks after SCI (Figure. 5A-B). The increase in microglia number was not restricted to the white matter, where Wallerian degeneration of descending axons injured at the epicenter is expected to provoke active inflammation at around this time point [28], but was also observed in the gray matter. Stereological counting showed a comparable number of microglial cells in the gray and white matter (Figure. 5G and 5H). The increase in the number of microglia was not affected by TMT in either gray or white matter.

**Figure 6:**
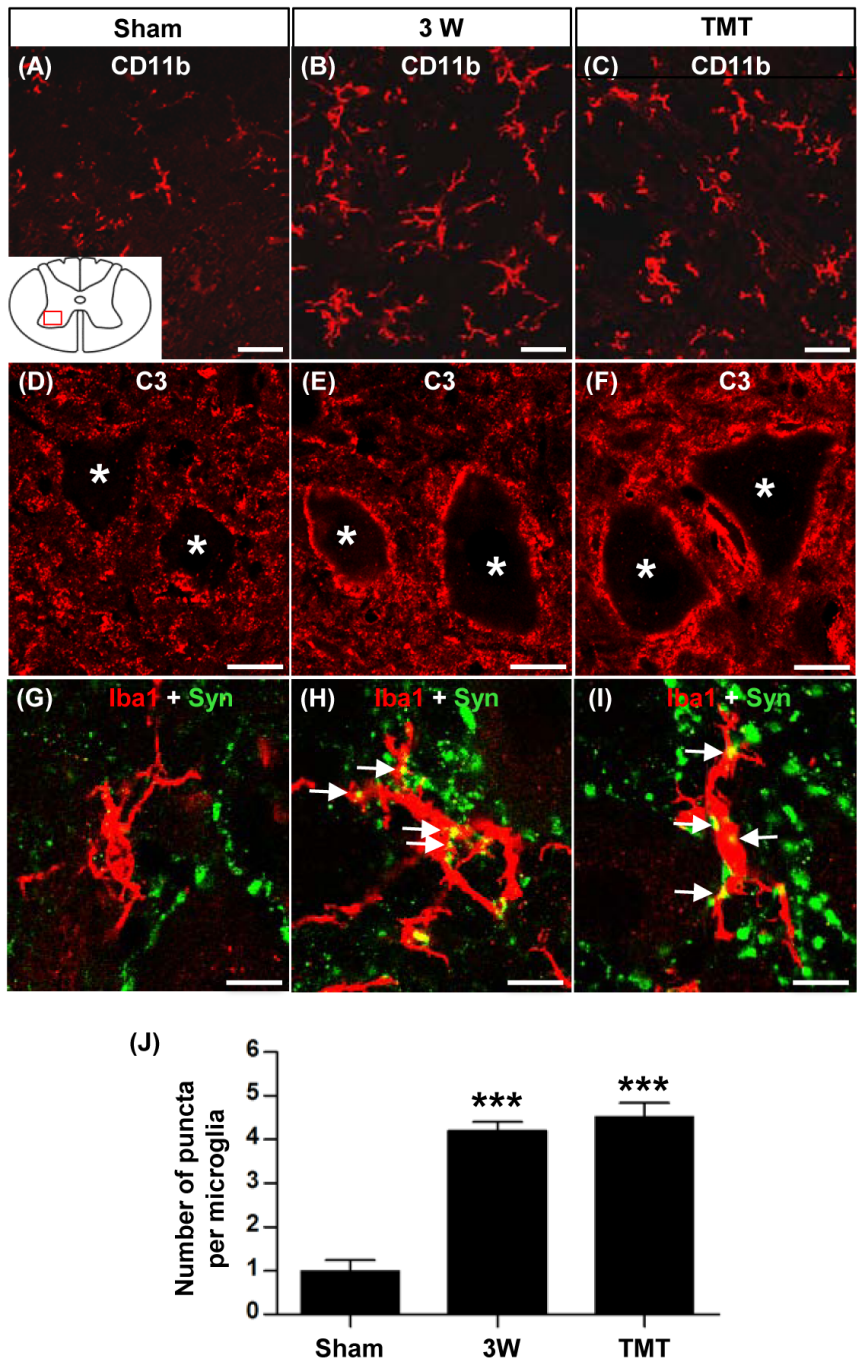
Involvement of microglial cells in synaptic remodeling in the lumbar motor region following thoracic spinal cord injury. (A-I) Representative images of transverse spinal cord sections from sham operated animals (A, D, G), animals at 3 weeks (3W) after injury (B, E, H), and animals at 3W after injury with treadmill training (TMT) (C, F, I). (A-C) Sections were stained with antibodies against CD11b (subunit of complement receptor 3). (D-F) Sections were stained with antibodies against C3 (complement component 3). Asterisks indicate cell bodies of ventral motor neurons. (G-I) Representative images of sections colabeled with Iba1 (red) and synaptotagmin (Syn; green). Each image consists of only a single optical plane. Double-labeled punctate signals (yellow) were considered as engulfed synaptic puncta (indicated by white arrows). The diagram inset in (A) shows the approximate location of regions of interest (red-boxed region within the ventral horn) for all the images. All scale bars represent 10 µm. (J) Quantification of the number of colocalized synaptic puncta per microglia. N = 5 animals for each group. *** represents *p*<0.001 by one-way ANOVA followed by Tukey's post-hoc analysis. Error bars represent SEM.

In the ventral gray matter at the lumbar level, Iba1-positive microglial cells were located near MAP2-positive neuronal cell bodies or dendritic neuropil (Figure. 5D-F), suggesting that intimate interactions between neurons and microglia could occur. Several studies have demonstrated roles of microglia in remodeling synaptic circuits during development or disease [29]–[31]. The complement cascade has also been implicated in synaptic remodeling [32], and a recent study showed that the interaction between neuronal C3 and microglial CR3 mediates the engulfment of synaptic puncta during development [29]. The microglial cells located in the ventral gray matter were positive for another CR3 component, CD11b (Figure. 6A and 6B). We also observed increased C3 protein around neuronal cell bodies and in the neuropil areas (Figure. 6D and 6E). Therefore, we tested the possibility that increased microglial activity in the ventral gray matter is involved in the removal of synaptic structures following SCI at the thoracic level. Synaptotagmin immunopositive puncta colocalizing with Iba1 immunoreactivity were considered as engulfed presynaptic material (Figure. 6G). The number of engulfed presynaptic puncta was almost three times higher at 3 week after injury in animals with SCI than in sham-operated controls (Figure. 6H and 6J). TMT for 2 weeks did not influence the expression of CR3 in microglia or C3 in neurons, or the number of engulfed synaptotagmin positive puncta (Figure. 6C, 6F, 6I, and 6J), suggesting that the potential synaptic remodeling mediated by microglia is unlikely to be regulated by TMT.

### Regulation of the injury-induced gene expression profiles by TMT

To examine the effects of TMT on gene expression after injury, expression profiles were compared at 3 weeks after injury in animals with and without TMT. To exclude genes that are regulated by TMT independently of SCI, we compared gene expressions between sham operated animals with and without TMT. A total of 146 genes (60 upregulated and 86 downregulated) were regulated by TMT irrespective of SCI (Table S4). Five out of the 146 gene were found to be differentially expressed between animals at 3 weeks after injury with and without TMT (1 upregulated and 4 downregulated). Therefore, these 5genes were excluded from the analysis on TMT-regulated genes in the lumbar spinal cord after SCI.

**Figure 7:**
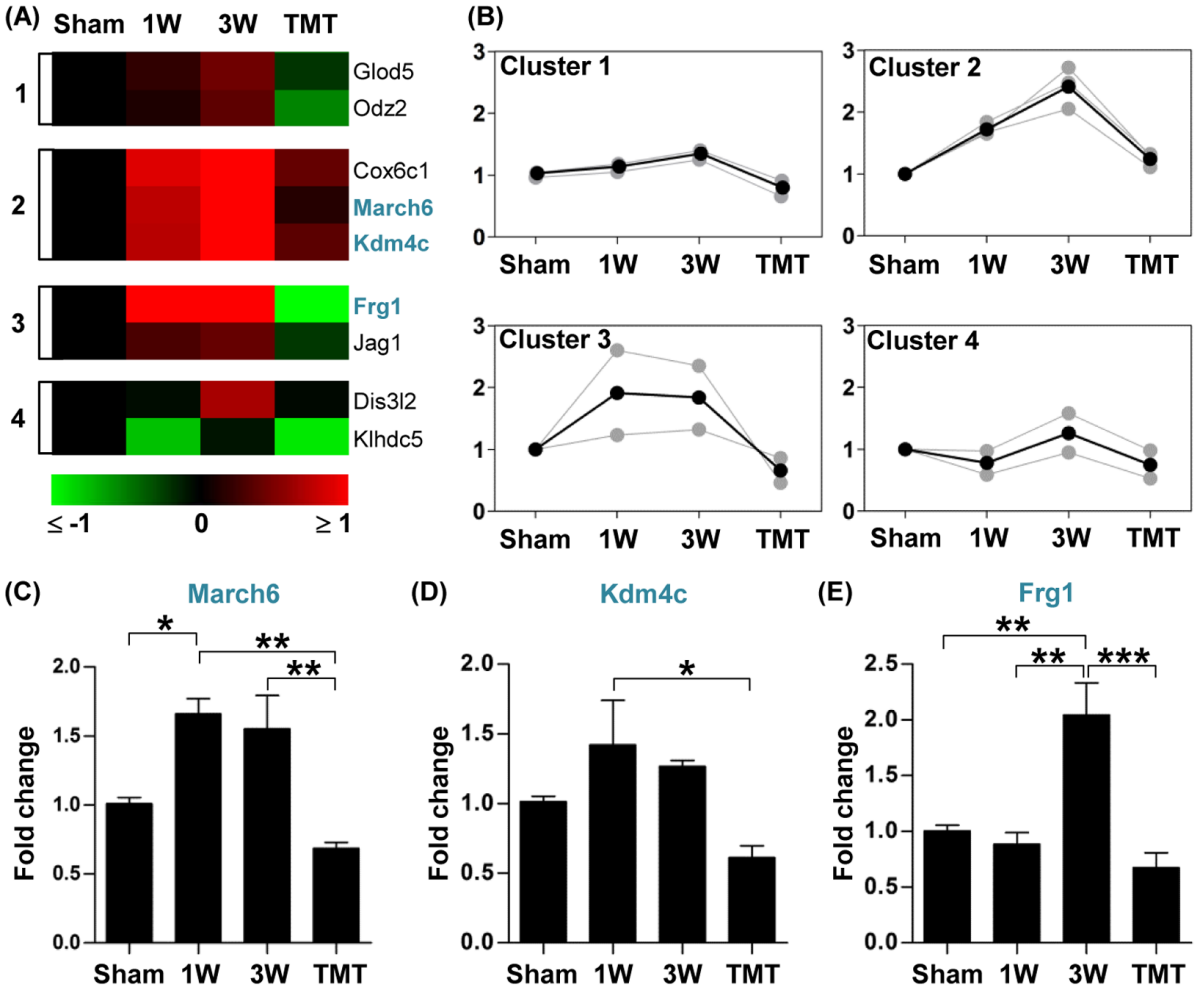
Genes downregulated by treadmill training. (A) Color-coded heatmaps of gene expression levels for genes whose expression was lower with treadmill training (TMT) than without TMT at 3 week after injury. Fold changes in gene expression level relative to the sham group expression level were log_2_-transformed and color-coded based on the color scale shown at the bottom. Genes were grouped into 4 clusters using a k-means clustering algorithm. The numbers shown at the left side of the heatmaps indicate the cluster indexes to which the genes belong. Genes for which validation data are represented are shown in blue. (B) Expression patterns of genes in each cluster. Gray lines indicate expression levels of individual genes and black lines indicate the average expression level of all genes in the cluster. Numbers on the Y-axis indicate log_2_-transformed fold changes relative to the expression level in the sham group. (C-E) Graphs of real-time RT-PCR results for genes selected from the list in (A) (shown in blue). N = 4 animals for each group. *, **, and *** represent *p*<0.05, *p*<0.01, and *p*<0.001, respectively, by one-way ANOVA followed by Tukey's post-hoc analysis. Error bars represent SEM.

Compared to the gene expression level at 3 weeks after injury, 9 genes were significantly downregulated in animals subjected to the 2-week TMT protocol (from 1 to 3 week). These genes are related to transcriptional activity, protein degradation, and cell-to-cell communication (Table 4). A much larger number of genes were upregulated by TMT; the expression of 49 genes was significantly upregulated in animals with TMT compared to those without TMT. The majority of these upregulated genes are involved in transcriptional activity, metabolism and biosynthesis, intracellular signaling, synapse, and angiogenesis (Table 4). Using the k-means clustering algorithm, genes regulated by TMT were classified into different clusters based on changes in gene expression pattern by injury and TMT. Of the downregulated genes, the majority belonged to cluster 1, 2 or 3, in which gene expression levels tended to increase after injury but to decrease with TMT (Figure. 7A and 7B). We chose three genes in these clusters and verified the microarray results by real-time RT-PCR. The expression of the March6 gene, encoding a ring finger family E3 ubiquitin ligase, was increased at 1 and 3 weeks after SCI, but TMT reversed the expression to the pre-injury level (Figure. 7C). An epigenetic modifier Kdm4c (also known as Jmjd2c), which possesses histone demethylase activity (H3K9) [33], showed a similar pattern of gene expression (Figure. 7D). The Frg1 gene is inappropriately overexpressed in facioscapulohumeral muscular dystrophy [34] and thought to function in mRNA splicing [35]. Frg1 showed robust upregulation at the 3-week time point and complete normalization by TMT (Figure. 7E).

**Table 4:** A list of genes that were regulated by treadmill locomotor training.

Functional group	Gene symbol	Gene full name
Cell communication	Jag1	jagged1
	Odz2	teneurin transmembrane protein 2
Ubiquitination & proteosome	March6	membrane-associated ring finger (C3HC4) 6
	Klhdc5	kelch domain containing 5
Transcription & epigenetics	Frg1	FSHD regions gene 1
	Kdm4c	lysine (K)-specific demethylase 4C (Jmjd2c)
Metabolism	Glod5	glyoxalase domain containing 5
	Cox6c-ps1	cytochrome c oxidase subunit VIc, pseudogene
Proliferation	Dis3l2	DIS3 mitotic control homolog (S. cerevisiae)-like 2
Immune & inflammation	Stat5b	signal transducer and activator of transcription 5B
	Icoslg	inducible T-cell co-stimulator ligand
	Crlf2	cytokine receptor-like factor 2
Intracellular signaling	Grb10	growth factor receptor bound protein 10
	Arhgap32	Rho GTPase activating protein 32
	Arfgef2	ADP-ribosylation factor guanine nucleotide-exchange factor 2 (brefeldin A-inhibited)
	Pde2a	phosphodiesterase 2A, cGMP-stimulated
	Farp2	FERM, RhoGEF and pleckstrin domain protein 2
	Tbc1d10b	TBC1 domain family, member 10b
	Raph1	ras association (RalGDS/AF-6) and pleckstrin homology domains 1
	Fkbp14	FK506 binding protein 14
	Ppp1r1b	protein phosphatase 1, regulatory (inhibitor) subunit 1B
Transcription & DNA repair	Fubp3	far upstream element (FUSE) binding protein 3
	Usf2	upstream transcription factor 2, c-fos interacting
	Pias1	protein inhibitor of activated STAT, 1
	Aqr	aquarius homolog (mouse)
	Mkl1	megakaryoblastic leukemia (translocation) 1
	Ints3	integrator complex subunit 3
	Zfp709	zinc finger protein 709
	Polb	polymerase (DNA directed), beta
Synapse	Nrcam	neuronal cell adhesion molecule
	Arc	activity-regulated cytoskeleton-associated protein
	Nlgn1	neuroligin 1
	Gja4	gap junction protein, alpha 4 (connexin-37)
Metabolism & biosynthesis	Retsat	retinol saturase (all trans retinol 13,14 reductase)
	Mtmr1	myotubularin related protein 1
	Slc13a3	solute carrier family 13 (sodium-dependent dicarboxylate transporter), member 3
	Chst12	carbohydrate (chondroitin 4) sulfotransferase 12
	Immt	inner membrane protein, mitochondrial
	Cyb5a	cytochrome b5 type A (microsomal)
	Bckdha	branched chain ketoacid dehydrogenase E1, alpha polypeptide
	Bcat1	branched chain amino acid transaminase 1, cytosolic
Angiogenesis	Adam8	ADAM metallopeptidase domain 8
	Tie1	tyrosine kinase with immunoglobulin-like and EGF-like domains 1
Apoptosis	Stk17b	serine/threonine kinase 17b (Drak2)
Translation	Gatc	glutamyl-tRNA(Gln) amidotransferase, subunit C
	Zc3h14	zinc finger CCCH-type containing 14
Cytoskeleton	Epb4.1	erythrocyte protein band 4.1
	Capg	capping protein (actin filament), gelsolin-like
	Myo1e	myosin 1E
Lysosome	Laptm4a	lysosomal protein transmembrane 4 alpha
Ubiquitination & proteosome	Ngly1	N-glycanase 1 (PNGase)
	Dcaf8	DDB1 and CUL4 associated factor 8 (Wdr42a)
Proliferation	Spag5	sperm associated antigen 5
Neuropeptide	Sst	Somatostatin
Undetermined	Dd25	hypothetical protein Dd25
	Samd10	sterile alpha motif domain containing 10
	Trim26	tripartite motif-containing 26
	Rnase4	ribonuclease, RNase A family 4

**Figure 8:**
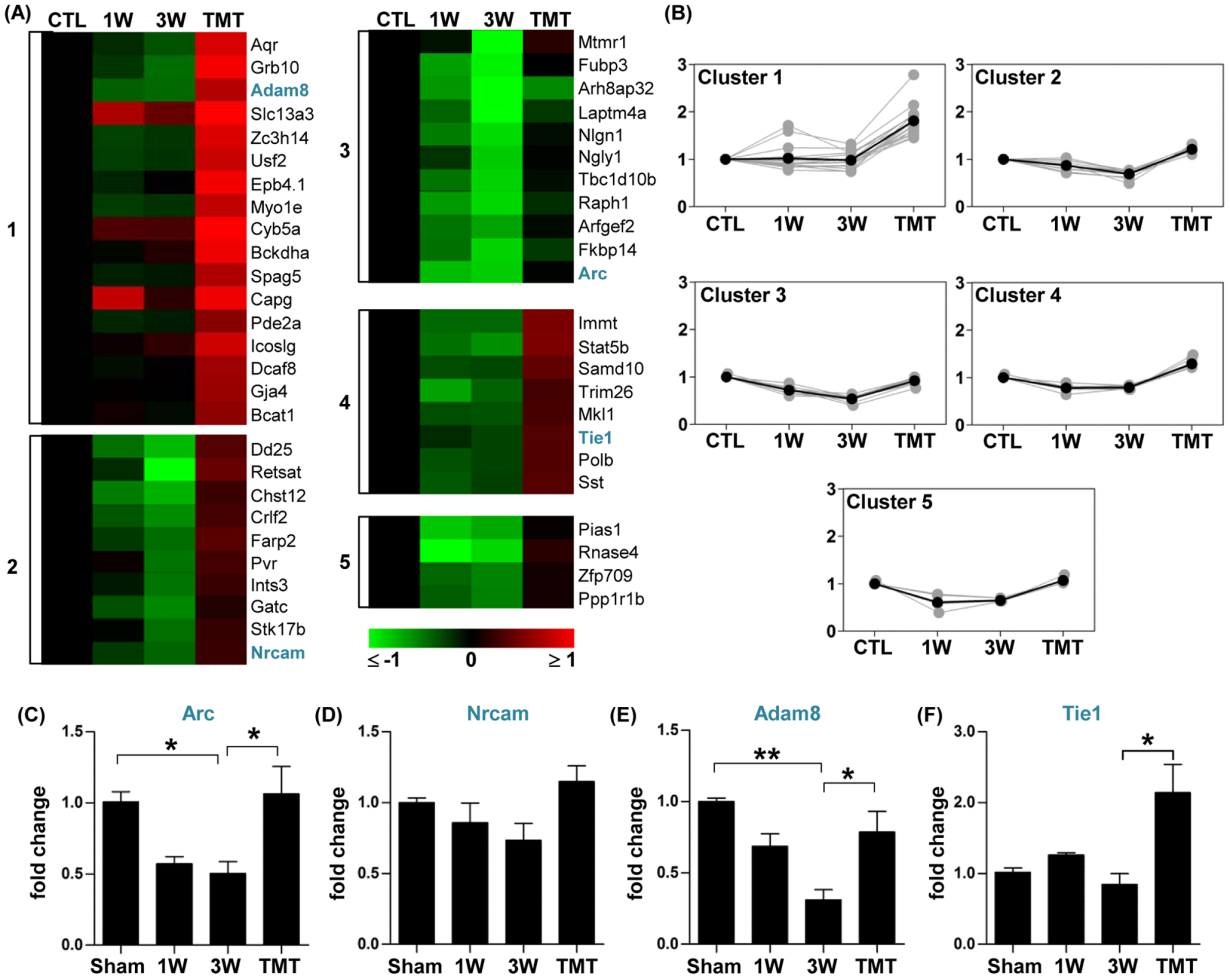
Genes upregulated by treadmill training. (A) Color-coded heatmaps of gene expression levels for the genes whose expression was higher with treadmill training (TMT) than without TMT at 3 weeks after injury. Fold changes in gene expression level relative to the sham group expression level were log_2_-transformed and color-coded based on the color scale shown at the bottom. Genes were grouped into 5 clusters using a k-means clustering algorithm. The numbers shown at the left side of heatmaps indicate the cluster indexes to which the genes belong. Genes for which validation data are presented are shown in blue. (B) Expression patterns of genes in each cluster. Gray lines indicate expression levels of individual genes and black lines indicate the average expression level of all genes in the cluster. Numbers on the Y-axis indicate log_2_-transformed fold changes relative to the expression level in the sham group. (C-F) Graphs of real-time RT-PCR results for genes selected from the list in (A) (shown in blue). N = 4 animals for each group. *, **, and *** represent *p*<0.05, *p*<0.01, and *p*<0.001, respectively, by one-way ANOVA followed by Tukey's post-hoc analysis. Error bars represent SEM.

Genes upregulated by TMT were classified into 5 clusters. The expression of genes in cluster 1 was not obviously changed by injury but markedly increased by TMT (Figure. 8A and 8B). A larger number of genes showed a variable degree of downregulation by injury with TMT inducing either reversal to the pre-injury level (clusters3 and 5) or an increase above the control value (clusters 2 and 4) (Figure. 8A and 8B). In general, TMT tended to upregulate the expression of genes in functional classes corresponding to the functional classes of genes that were downregulated by injury. For example, genes related to metabolism and biosynthesis were downregulated by injury (Figure. 2), and TMT upregulated 9 genes belonging to this functional category (Table 4). Genes with functions in synapse and angiogenesis were also downregulated by injury (Figure. 2). TMT upregulated synapse-related genes such as Arc, Nrcam, and Nlgn1. Similarly, the angiogenesis-related genes Tie1 and Adam8 were significantly upregulated by TMT.

**Figure 9:**
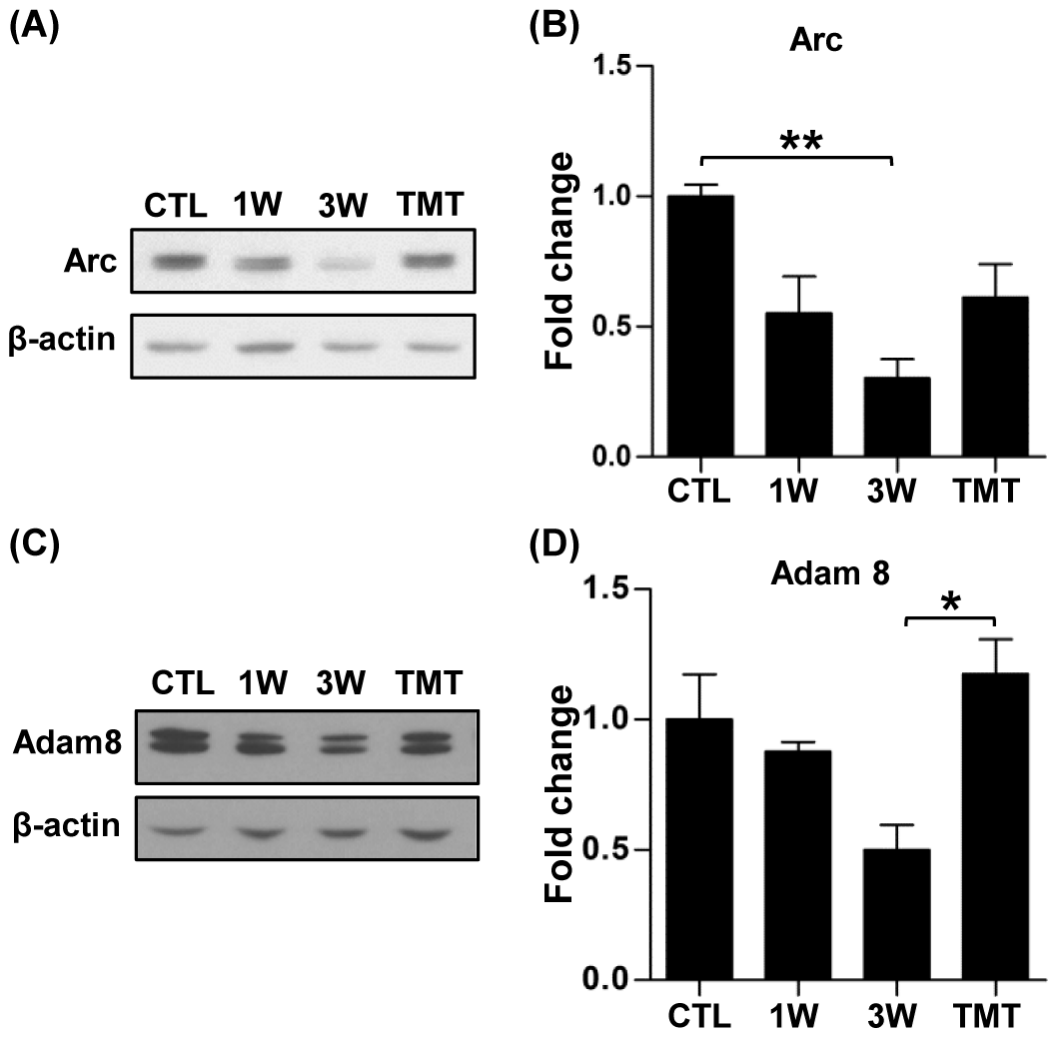
Validation of changes in protein expression from the Arc and Adam8 genes. (A, C) Representative western blots of Arc (A) and Adam8 (C). β-actin was used to normalize differences in loading amounts. (B, D) Quantification graphs of Arc (B) and Adam8 (D) western blots. Data are presented as fold-changes relative to the normal protein expression level after normalization with β-actin. N = 3 to 4 animals for each group. * and ** represent *p*<0.05 and *p*<0.01, respectively, by one-way ANOVA followed by Tukey's post-hoc analysis.

To confirm the trend of TMT-induced restoration of gene expression observed in the microarray experiments, a combination of real-time PCR and western blotting was used. First, the expression pattern of two of the genes related to synapse function, Arc and Nrcam (neural cell adhesion molecule), was examined. Arc is a well-characterized immediate-early gene with a function in homeostatic plasticity at the synapse [36]. Real-time RT-PCR showed that the level of Arc gene expression tended to decrease at 1 and 3 weeks after SCI, and TMT significantly increased the mRNA expression above the pre-injury level (Figure. 8C). Arc expression was also examined at the protein level. We found that the Arc protein level substantially decreased at 3 weeks after SCI and tended to get normalized by TMT (Figure. 9A and 9B). Real-time RT-PCR also showed that the Nrcam mRNA level tended to decrease at 3 weeks after SCI and to increase to slightly above the pre-injury level with TMT (Figure. 8D), consistent with the microarray result.

The TMT-induced changes in expression of two genes with functions in angiogenesis were also validated. Adam8 is selectively expressed in endothelial cells and is associated with angiogenesis at the epicenter in a mouse SCI model [37]. Tie1 is an endothelial specific tyrosine kinase and known to play a regulatory role in angiopoietin1-dependent angiogenesis [38]. Real-time RT-PCR revealed robust reduction of Adam8 gene expression at the 3-week time point (Figure. 8E). The Adam8 gene expression was substantially restored close to the pre-injury level with TMT. Consistent with the gene expression data, we found that Adam8 protein exhibited a marked decrease at 3 weeks, and TMT resulted in an increase of the Adam8 protein level (Figure. 9C and 9D). TMT-induced significant upregulation of the Tie1 gene was also verified by real-time RT-PCR (Figure. 8F).

## Discussion

In this study, we characterized the patterns of gene expression changes in the lumbar spinal cord following thoracic contusive SCI with or without TMT, an intervention to promote locomotor recovery. Our data showed robust upregulation of inflammation-related genes at both 1 and 3 weeks after injury. Consistent with the gene expression changes, the number of microglial cells in the lumbar motor region substantially increased. Further experiments suggested potential roles of the complement components and microglial cells in synaptic remodeling of the lumbar motor circuit. Comparison of gene expression profiles between animals with or without TMT showed that the upregulation of inflammation-related genes was not appreciably affected by TMT. However, TMT frequently restored the expression levels of genes that were downregulated by injury. Notably, TMT increased the expression of genes involved in neuroplasticity and angiogenesis, suggesting that promoting neurovascular remodeling may in part be a mechanism by which TMT contributes to locomotor recovery.

The microarray method has been employed in many studies to profile gene expression changes in the spinal cord following SCI [13]–[15], [39]–[44]. Most of these studies have focused on the gene expressions only at the epicenter or at the distal segment immediately adjacent to the epicenter where secondary tissue degeneration could be observed. To the authors’ knowledge, only one study has examined transcriptional changes in a region sufficiently caudal to the epicenter (T8 in that study) to include the lumbar locomotor circuit, although it was not explicitly stated whether they intended to sample the lumbar motor region [45]. The pattern of gene expression changes in our study was similar to that in the caudal segment at comparable time points after SCI reported in the study by Aimone et al. (2004). The majority of the transcriptome changes in their study were not specific to the caudal segment but followed a similar pattern at the epicenter and rostral segment, suggesting that the global trend of gene expression changes spread rostrocaudally irrespective of functional distinction at different segmental regions. It is worth reporting that expressions of genes known to be highly specific for the peripheral nerves (such as P0 and PMP22) were observed in our samples despite our efforts to remove the root components during the dissection of spinal cord tissues. A caveat remains, therefore, that some of gene expression profiles found in microarray may result from transcriptional changes in the peripheral nerve.

In our study, the most robust changes were observed in genes related to immune processes or inflammation, including genes involved in immune cell differentiation and proliferation, inflammatory signaling pathways, cytokines, and the complement system. As mentioned above, the increases in expression of inflammation-related genes observed in previous studies occurred diffusely along the spinal cord: in the epicenter, adjacent regions, and rostrocaudal segments quite distant from the epicenter [15], [45]. Therefore, the robust upregulation of inflammation-related genes observed in our study is unlikely to be specific to the caudal segment below the lesion site or the lumbar motor circuit.

A notable observation in our study was the robust and concurrent upregulation of the components of an early complement cascade leading to C3 activation (Figure. 3). Activation of the complement system around the injury site may facilitate phagocytic cell removal of dead cells or participation in the secondary degeneration of neural cells [46], [47]. In addition, formation of the membrane attack complex (C5b-9) may induce neural cell death [48], [49]. We observed persistent upregulation of the early complement pathway in the lumbar motor region where there is no overt tissue degeneration. In contrast, the complement components involved in the formation of membrane attack complex was not activated, whereas it was reported that they are strongly activated at the epicenter after SCI [49]. This suggests that the activation of the early complement pathway in the caudal region may serve functions different from those at the epicenter. Recent studies have suggested that activation of the complement system may promote elimination of synapses by microglial phagocytosis [29], [32]. Our data revealed microglial activation in the gray matter in close proximity to ventral motor neurons and their dendritic processes. We further showed that neurons in the ventral horn upregulated C3 and microglial cells in the vicinity of motor neurons were strongly positive for CD11b, a subunit of the CR3 receptor, which, upon binding of C3, stimulates phagocytic activity in microglial cells [26]. Therefore, we speculated that activated microglia near motor neurons after SCI may participate in the remodeling of synaptic structures in a manner similar to that in which they are involved in synaptic pruning during neural circuit formation [29]. Indeed, the number of synaptic puncta colocalizing with the microglial marker was substantially increased in the lumbar motor region after SCI. However, the synaptic pruning by microglia in the previous study was activity-dependent [29], whereas the extent of synapse elimination in our study was not affected by TMT, which supposedly increases neural activity of the locomotor circuit in the lumbar motor region. It remains to be determined whether the synaptic remodeling elicited by injury in the mature CNS is independent of activity or whether changes in neural activity in response to TMT were not strong enough to affect the removal of synaptic elements by microglia.

When the effects of TMT on gene expression after SCI were examined, TMT did not significantly influence the expression of inflammation-associated genes that were robustly upregulated SCI. However, a trend towards reversal of other SCI-induced gene expression changes was observed. A large number of genes that were downregulated after SCI were upregulated by TMT and vice versa (Figure. 7 and 8). The majority of TMT-induced changes were upregulations, implying that a principal molecular mechanism mediating TMT-induced locomotor recovery is to restore transcriptional activities of the genes of which expression levels are decreased by SCI. Intriguingly, however, we observed a distinct group of genes showing obvious upregulation by injury and normalization by TMT (Figure. 7). Overexpression of Frg1, a gene whose expression is altered in facioscapulohumeral muscular dystrophy, results in aberrant alternative pre-mRNA splicing associated with a muscular dystrophy phenotype in transgenic mice [35]. This suggests that high Frg1 gene expression after SCI may lead to a gain of dysfunction caused by altered transcription after abnormal splicing activity. In a similar vein, inappropriately augmented Kdm4c or March6 expression also may entail cellular dysfunction by dysregulating the methylation status of histones [33] or by altering ubiquitin-mediated proteolysis, respectively. TMT not only enhances locomotor recovery but also ameliorates dysfunctions including allodynia [50] and spasticity [51]. It is conceivable that normalizing inappropriate upregulation of these genes may contribute to the attenuation by TMT of such SCI-induced dysfunctions.

Gene expression profiling in the lumbar motor regions at 3 weeks revealed reduced transcriptional activities of genes related to neural functions (neural development, axon, synapse, neurotransmitter transport, etc), angiogenesis (including endothelial cell development), and metabolic processes (Figure. 2). These findings indicate that inadequate functions in these processes may in part be responsible for the limited extent of spontaneous locomotor recovery. In other words, restoring optimal functions in these processes may promote locomotor recovery following SCI. In agreement with this notion, TMT restored the expression of many genes involved in these processes to their pre-injury levels or boosted their expression to even higher than their control levels. These findings my provide clues as to potential mechanisms mediating TMT-induced locomotor recovery. Of the genes that were upregulated by TMT, we were particularly interested in genes involved in neuroplasticity and angiogenesis. Nrcam (neural cell adhesion molecule) has been shown to play a role in synaptic plasticity, neuritogenesis, and synaptogenesis [52]–[54]. Therefore, restoration of Nrcam expression by TMT may ensure an adequate level of plasticity after injury. Arc, also known as Arg3.1, is not only involved in plasticity of synaptic strength (long-term potentiation or depression) but also in structural plasticity of dendritic spines [55]–[58]. Therefore, a decrease in the basal Arc level may lead to dysregulated spine remodeling occurring caudal to the injury level [59]. Furthermore, Arc has been implicated in regulating the homeostatic plasticity that maintains the neuronal threshold of excitability in response to chronic changes in synaptic activity [36]. For example, a loss of Arc leads to excess network hyperexcitability [58]. Decreased expression of Arc in the lumbar motor regions after SCI may contribute in part to the dysregulated spinal cord excitability that is responsible for functional disabilities observed in patients with SCI [60], [61]. Together, this suggests that restoration of the level of Arc by TMT may contribute to improvement of locomotor function via multiple mechanisms.

TMT also upregulated the expression of the angiogenesis-related genes Adam8 and Tie1. It was recently reported that Adam8 is upregulated selectively in endothelial cells in the spinal cord and may regulate angiogenesis after SCI [37]. In particular, Adam8 may play a role in neovascularization by regulating ectodomain shedding of membrane proteins related to angiogenesis [62], [63]. Tie1 is known to be an important regulator of angiopoietin-1 signaling in angiogenesis and vascular maintenance [64]. Based on these known functions, TMT-induced upregulation of Adam8 and Tie1 may promote vascular remodeling in the lumbar motor regions after SCI. A previous *in vivo* imaging study demonstrated a dynamic vascular remodeling in the lesioned spinal cord [65]. It is conceivable that a similar vascular remodeling process occurs in the lumbar motor region below the lesion, and that the vascular remodeling is one of the principal biological processes that are substantially influenced by TMT.

Together, our data showing that TMT concurrently upregulates neuroplasticity genes and angiogenesis genes suggests that TMT may contribute to locomotor recovery through promotion of neurovascular remodeling in the lumbar motor region. This notion is particularly intriguing because neural circuit formation and angiogenesis are thought to share common mechanisms [66]. It is possible that this shared signaling during development may be reactivated following injury. Indeed, dynamic neurovascular interactions have been observed in lesioned spinal cord [65]. Further studies will be needed to identify these developmentally linked signals that regulate neurovascular remodeling in the lumbar motor region thereby mediating locomotor recovery following SCI.

## Supporting Information

Table S1 A list of genes that were differentially expressed only at 1-week time point.
(PDF)

Table S2 A list of genes that were differentially expressed only at 3-week time point.
(PDF)

Table S3 A list of genes that were differentially expressed only at 3-week time point.
(PDF)

Table S4 A list of genes that were differentially regulated by treadmill training in sham- operated animals.
(PDF)
