# Supplementary material for: Molecular and Cellular Changes in the Lumbar Spinal Cord following Thoracic Injury: Regulation by Treadmill Locomotor Training
Source: PLoS One. 2014 Feb 10;9(2):e88215. doi: 10.1371/journal.pone.0088215 (PMC3919755; doi:10.1371/journal.pone.0088215)
Supplement: Table S1 — A list of genes that were differentially expressed only at 1-week time point. (PDF) [file pone.0088215.s001.pdf]

Table S1. A list of genes that were differentially expressed only at 1-week time point

Probe ID	Gene symbol	Gene full name	Log2 (fold change)	*p* Value
**Upregulated genes**				
1378640_at	Uhrf1	ubiquitin-like with PHD and ring finger domains 1	2.12	0.04069
1377146_at	Vip	vasoactive intestinal peptide	1.96	0.03927
1393041_at	Smc2	structural maintenance of chromosomes 2	1.74	0.03731
1385213_at	Epsti1	epithelial stromal interaction 1 (breast)	1.59	0.00736
1389011_at	Nsmce2	non-SMC element 2, MMS21 homolog (S. cerevisiae)	1.54	0.01602
1368327_at	Slc12a9	solute carrier family 12 (potassium/chloride transporters), member 9	1.42	0.03358
1369047_at	Sult1d1	sulfotransferase family 1D, member 1	1.36	0.00504
1395715_at	Cdgap	cdc42 GTPase-activating protein	1.32	0.01704
1373523_at	Fcgr3a	Fc fragment of IgG, low affinity IIIa, receptor	1.29	0.04672
1369290_at	Ccr5	chemokine (C-C motif) receptor 5	1.27	0.00809
1376377_at	LOC290396	similar to Diaph3 diaphanous homolog 3 (Drosophila)	1.26	0.00661
1371541_at	Mylk	myosin light chain kinase	1.18	0.04508
1391108_at	Znf593	zinc finger protein 593	1.13	0.02540
1387270_at	Hhex	hematopoietically expressed homeobox	1.13	0.02310
1389425_at	LOC688261	similar to 5(3)-deoxyribonucleotidase, cytosolic type (Cytosolic 5,3-pyrimidine nucleotidase) (Deoxy-5-nucleotidase 1) (dNT-1)	1.10	0.03889
1383377_at	Gabpa	GA binding protein transcription factor, alpha subunit	1.10	0.04734
1389568_at	Calhm2	calcium homeostasis modulator 2	1.08	0.03147
1390830_at	LOC499602	hypothetical protein LOC499602	1.08	0.02668
1388157_at	Marcks	myristoylated alanine rich protein kinase C substrate	1.08	0.01160
1383271_at	Ccdc59	coiled-coil domain containing 59	1.07	0.03619
1389253_at	Vnn1	vanin 1	1.04	0.04761
1389838_at	LOC681252	similar to myristoylated alanine-rich C-kinase substrate (MARCKS)	1.04	0.00805
1388157_at	LOC294446	similar to myristoylated alanine-rich C-kinase substrate (MARCKS) (ACAMP-81)	1.04	0.00805
1380517_at	Zmym3	zinc finger, MYM-type 3	1.04	0.00907
1391864_at	Enpp6	ectonucleotide pyrophosphatase/phosphodiesterase 6	1.02	0.03585
1368754_at	P2ry6	pyrimidinergic receptor P2Y, G-protein coupled, 6	1.00	0.00205
1367570_at	Tagln	transgelin	0.99	0.02559
1382874_at	LOC304860	N-acetylneuraminate pyruvate lyase	0.93	0.00792
1386925_at	Arpc1b	actin related protein 2/3 complex, subunit 1B	0.92	0.03370
1397620_at	Sh3rf1	SH3 domain containing ring finger 1	0.91	0.00889
1393038_at	Fcgr1a	Fc fragment of IgG, high affinity Ia, receptor (CD64)	0.91	0.02190
1382206_a_at	Akap2	A kinase (PRKA) anchor protein 2	0.91	0.01535
1394999_at	RGD1562617	similar to RAB7-like protein	0.90	0.00257
1370139_a_at	Trpc6	transient receptor potential cation channel, subfamily C, member 6	0.90	0.04455
1369157_at	Pde3b	phosphodiesterase 3B, cGMP-inhibited	0.89	0.02056
1379659_at	Bmp2k	BMP-2 inducible kinase	0.88	0.02213
1383914_at	LOC286991	putative retrovirus-related gag protein	0.88	0.00353
1383419_at	Lhfpl3	lipoma HMGIC fusion partner-like 3	0.88	0.00448
1379475_at	RGD1563120	similar to RIKEN cDNA 2210009G21	0.86	0.03588
1376411_at	LOC304037	ADP-ribosylation factor-like 13B	0.86	0.04224
1373546_at	Atp11c	ATPase, class VI, type 11C	0.85	0.04039
1393459_at	Fmr1	fragile X mental retardation 1	0.84	0.01817
1393201_at	Lrmp	lymphoid-restricted membrane protein	0.83	0.00484
1370045_at	Polg	polymerase (DNA directed), gamma	0.82	0.00049
1387248_at	Kcnj5	potassium inwardly-rectifying channel, subfamily J, member 5	0.82	0.01355
1376858_at	LOC686809	similar to protein 7 transactivated by hepatitis B virus X antigen	0.82	0.04302
1367723_a_at	Sh2b3	SH2B adaptor protein 3	0.81	0.02486
1373785_at	LOC362792	phospholipase D family, member 4	0.81	0.04507
1397471_at	LOC690976	similar to Calponin-2 (Calponin H2, smooth muscle)	0.80	0.02395
1368280_at	Ctsc	cathepsin C	0.80	0.01798
1369006_at	Hk2	hexokinase 2	0.79	0.03270
1391475_at	Hnrpll	heterogeneous nuclear ribonucleoprotein L-like	0.78	0.04529
1384500_at	RGD1305604	coiled-coil domain containing 30	0.78	0.03672
1385426_at	Ccdc109b	coiled-coil domain containing 109B	0.77	0.04720
1368490_at	Cd14	cd14 molecule	0.77	0.03440
1383319_at	Slc4a10	solute carrier family 4, sodium bicarbonate transporter-like, member 10	0.76	0.03901
1387247_at	Pcsk1	proprotein convertase subtilisin/kexin type 1	0.76	0.01450
1395015_at	F8	coagulation factor VIII, procoagulant component	0.76	0.03143
1378003_at	Lrrc8b	leucine rich repeat containing 8 family, member B	0.76	0.01201
1376591_a_at	LOC690263	transmembrane protein 179B	0.76	0.01555
1379232_at	LOC686471	hypothetical protein LOC686471	0.76	0.01555
1378032_at	Nfkbiz	nuclear factor of kappa light polypeptide gene enhancer in B-cells inhibitor, zeta	0.75	0.04500
1388711_at	Il13ra1	interleukin 13 receptor, alpha 1	0.75	0.04098
1396123_at	Tctex1d2	tctex1 domain containing 2	0.75	0.02465
1374281_at	Ino80	ino80 homolog (S. cerevisiae)	0.74	0.01953
1369956_at	Ifngr1	interferon gamma receptor 1	0.74	0.01666
1376039_at	Aurka	aurora kinase A	0.74	0.00274
1372846_at	Cybasc3	cytochrome b, ascorbate dependent 3	0.73	0.03960
1374134_at	Elk3	elk3, member of ETS oncogene family	0.73	0.02401
1383895_at	Dynlrb2	dynein light chain roadblock-type 2	0.72	0.02030
1394787_at	RGD1309138	similar to hypothetical protein MGC9912	0.72	0.03353
1376144_at	Parp9	poly (ADP-ribose) polymerase family, member 9	0.71	0.00628
1379075_at	Mboat2	membrane bound O-acyltransferase domain containing 2	0.70	0.04333
1373262_at	RGD1309543	similar to 2310014H01Rik protein	0.70	0.04165
1389643_at	Wiz	widely-interspaced zinc finger motifs	0.70	0.02907
1383988_at	LOC679150	hypothetical protein LOC679150	0.70	0.01369
1374544_at	Mgc112715	platelet receptor Gi24	0.70	0.01369
1378225_at	Mmp14	matrix metallopeptidase 14 (membrane-inserted)	0.69	0.04126
1392592_at	LOC679869	similar to transcription factor 7-like 2, T-cell specific, HMG-box	0.69	0.01177
1369242_at	Pax6	paired box 6	0.68	0.01445
1370914_at	Dnttip2	deoxynucleotidyltransferase, terminal, interacting protein 2	0.68	0.02536
1372706_at	Hexb	hexosaminidase B	0.68	0.00516
1390312_at	LOC500015	sterile alpha motif domain containing 9-like	0.68	0.02814
1373421_at	RGD1560115	tgfb-induced factor homeobox 2	0.68	0.04655
1388015_at	Ptprz1	protein tyrosine phosphatase, receptor-type, Z polypeptide 1	0.67	0.02838

Probe ID	Gene symbol	Gene full name	Log2 (fold change)	*p* Value
1368742_at	C5ar1	complement component 5a receptor 1	0.67	0.03182
1387434_at	Slc22a4	solute carrier family 22 (organic cation transporter), member 4	0.67	0.01557
1387566_at	Pla2g4a	phospholipase A2, group IVA (cytosolic, calcium-dependent)	0.67	0.01063
1384954_at	Hs3st3b1	heparan sulfate (glucosamine) 3-O-sulfotransferase 3B1	0.67	0.02365
1377886_at	Trim6	tripartite motif-containing 6	0.66	0.00520
1386027_at	Rp2h	retinitis pigmentosa 2 homolog (human)	0.66	0.04596
1373273_at	Prpf38a	prp38 pre-mRNA processing factor 38 (yeast) domain containing A	0.66	0.03141
1390784_at	Wdr36	wd repeat domain 36	0.66	0.03981
1373391_at	Specc1	sperm antigen with calponin homology and coiled-coil domains 1	0.65	0.02197
1390048_at	LOC685890	serine/arginine repetitive matrix 3	0.65	0.02412
1387327_at	Khdrbs2	KH domain containing, RNA binding, signal transduction associated 2	0.64	0.04423
1383915_at	LOC686184	similar to Putative deoxyribose-phosphate aldolase (Phosphodeoxyriboaldolase) (Deoxyriboaldolase) (DERA)	0.64	0.02880
1375954_at	S100a13	s100 calcium binding protein A13	0.63	0.01298
1387513_at	Cyth3	cytohesin 3	0.63	0.00877
1374487_at	Fam96a	family with sequence similarity 96, member A	0.63	0.02104
1391766_at	Hs3st5	heparan sulfate (glucosamine) 3-O-sulfotransferase 5	0.63	0.02104
1389771_at	LOC681217	zinc finger protein 862	0.62	0.03591
1379295_at	LOC690825	guanine nucleotide binding protein (G protein), gamma transducing activity polypeptide 2	0.62	0.02077
1394006_at	Psme1	proteasome (prosome, macropain) activator subunit 1	0.62	0.00298
1369725_at	Adap2	ArfGAP with dual PH domains 2	0.62	0.01856
1392326_at	Dnah12	dynein, axonemal, heavy chain 12	0.62	0.02806
1384035_at	LOC686794	similar to liver-specific bHLH-Zip transcription factor	0.61	0.02252
1379320_at	Snrpd1	small nuclear ribonucleoprotein D1	0.61	0.04006
1390797_at	Lcp2	lymphocyte cytosolic protein 2	0.61	0.01003
1384254_at	Otud1	OTU domain containing 1	0.61	0.01411
1381836_at	LOC689377	member RAS oncogene family	0.61	0.01852
1390887_at	RGD1309995	similar to CG13957-PA	0.60	0.00955
1373860_at	Sox4	SRY (sex determining region Y)-box 4	0.60	0.00121
1374842_at	Cebpz	CCAAT/enhancer binding protein zeta	0.60	0.04015
1373817_at	Ing5	inhibitor of growth family, member 5	0.60	0.01615
1387946_at	Lgals3bp	lectin, galactoside-binding, soluble, 3 binding protein	0.59	0.04903
1370078_at	LOC360888	similar to lin-9 homolog (C. elegans)	0.59	0.00781
1383290_at	Spint1	serine peptidase inhibitor, Kunitz type 1	0.59	0.03930
1387659_at	Gda	guanine deaminase	0.59	0.01482
1368851_at	Ets1	v-ets erythroblastosis virus E26 oncogene homolog 1 (avian)	0.59	0.02888
**Downregulated genes**				
1387406_at	Uhmk1	U2AF homology motif (UHM) kinase 1	-1.48	0.02802
1373300_at	RGD1306151	similar to hypothetical protein DKFZp761D0211	-1.27	0.03575
1395074_at	Tdg	thymine-DNA glycosylase	-1.15	0.03463
1397867_at	Akap6	A kinase (PRKA) anchor protein 6	-1.15	0.04339
1369168_a_at	Clock	clock homolog (mouse)	-1.14	0.04725
1390812_a_at	Rerg	RAS-like, estrogen-regulated, growth-inhibitor	-1.10	0.03275
1369517_at	Cyth1	cytohesin 1	-1.09	0.04121
1381300_at	RGD1562390	similar to RGD, leucine-rich repeat, tropomodulin and proline-rich containing protein	-1.09	0.04670
1373046_at	Boll	bol, boule-like (Drosophila)	-1.07	0.02265
1369117_at	Calca	calcitonin-related polypeptide alpha	-1.07	0.02481
1369129_at	Rasgrp1	RAS guanyl releasing protein 1 (calcium and DAG-regulated)	-1.03	0.01424
1367753_at	Sec31a	SEC31 homolog A (S. cerevisiae)	-1.03	0.01292
1389774_at	Znf23	zinc finger protein 23 (KOX 16)	-1.02	0.04429
1374664_at	Znf787	zinc finger protein 787	-0.95	0.02634
1398108_at	Arhgdia	rho GDP dissociation inhibitor (GDI) alpha	-0.92	0.00375
1370233_at	Fgf12	fibroblast growth factor 12	-0.92	0.04584
1374654_at	Btrc	beta-transducin repeat containing	-0.92	0.03578
1367591_at	Prdx3	peroxiredoxin 3	-0.88	0.00033
1372968_at	Sbf1	SET binding factor 1	-0.88	0.04781
1392883_at	RGD1305269	Limch1 LIM and calponin homology domains 1	-0.87	0.01744
1389844_at	Fkbp4	FK506 binding protein 4	-0.84	0.04627
1369022_at	Svop	SV2 related protein	-0.82	0.02471
1371810_at	Copg	coatomer protein complex, subunit gamma	-0.81	0.01908
1388475_at	LOC313535	ERI1 exoribonuclease family member 3	-0.78	0.04743
1395405_at	Snx26	sorting nexin 26	-0.76	0.00748
1381971_at	Sox18	SRY (sex determining region Y)-box 18	-0.76	0.04714
1386846_at	Rprd1a	regulation of nuclear pre-mRNA domain containing 1A	-0.75	0.04245
1370982_at	Pygm	phosphorylase, glycogen, muscle	-0.75	0.04988
1391698_at	Mast1	microtubule associated serine/threonine kinase 1	-0.75	0.00470
1371578_at	Prkaca	protein kinase, cAMP-dependent, catalytic, alpha	-0.74	0.01829
1368117_at	Gphn	gephyrin	-0.72	0.04307
1371704_at	Ddx23	DEAD (Asp-Glu-Ala-Asp) box polypeptide 23	-0.72	0.05000
1371979_at	Srebf2	sterol regulatory element binding transcription factor 2	-0.72	0.00217
1380511_at	Parvb	parvin, beta	-0.71	0.03011
1398270_at	Bmp2	bone morphogenetic protein 2	-0.70	0.02571
1368807_at	Rtn3	reticulon 3	-0.69	0.04641
1372289_at	Slc25a12	solute carrier family 25 (mitochondrial carrier, Aralar), member 12	-0.67	0.01109
1368201_at	Npr1	natriuretic peptide receptor A/guanylate cyclase A (atrionatriuretic peptide receptor A)	-0.67	0.03851
1389906_at	Fdft1	farnesyl diphosphate farnesyl transferase 1	-0.67	0.00274
1387429_at	Adrbk1	adrenergic, beta, receptor kinase 1	-0.66	0.04318
1367959_a_at	Scn1b	sodium channel, voltage-gated, type I, beta	-0.65	0.02330
1370011_at	Ak1	adenylate kinase 1	-0.65	0.02942
1372310_at	Isoc1	isochorismatase domain containing 1	-0.65	0.01190
1371606_at	Wdtc1	WD and tetratricopeptide repeats 1	-0.64	0.04264
1371497_at	Asb6	ankyrin repeat and SOCS box-containing 6	-0.64	0.00419
1373971_at	Lpcat4	lysophosphatidylcholine acyltransferase 4	-0.64	0.00250
1387926_at	Sc5dl	sterol-C5-desaturase (ERG3 delta-5-desaturase homolog, S. cerevisiae)-like	-0.64	0.01887
1378297_at	RGD1305725	similar to chromosome 20 open reading frame 102	-0.63	0.04891
1387655_at	LOC683938	similar to stromal cell derived factor receptor 1	-0.63	0.04891
1389540_at	LOC683475	similar to IQ motif and Sec7 domain 2	-0.63	0.02538
1395534_at	Rasl10b	RAS-like, family 10, member B	-0.62	0.03636

Probe ID	Gene symbol	Gene full name	Log2 (fold change)	*p* Value
1378535_at	Phf7	PHD finger protein 7	-0.62	0.00977
1397806_at	LOC293494	ino80 complex subunit E	-0.62	0.01151
1367649_at	Palm	paralemmin	-0.61	0.02135
1367993_at	Clip1	CAP-GLY domain containing linker protein 1	-0.61	0.02374
1385124_at	Trib2	tribbles homolog 2 (Drosophila)	-0.59	0.00985
1368352_at	Stx1b	syntaxin 1B	-0.59	0.00847
1389064_at	Fem1c	fem-1 homolog c (C. elegans)	-0.59	0.01455
