# Supplementary material for: Molecular and Cellular Changes in the Lumbar Spinal Cord following Thoracic Injury: Regulation by Treadmill Locomotor Training
Source: PLoS One. 2014 Feb 10;9(2):e88215. doi: 10.1371/journal.pone.0088215 (PMC3919755; doi:10.1371/journal.pone.0088215)
Supplement: Table S2 — A list of genes that were differentially expressed only at 3-week time point. (PDF) [file pone.0088215.s002.pdf]

Table S2. A list of genes that were differentially expressed only at 3-week time point

Probe ID	Gene symbol	Gene full name	Log2 (fold change)	*p* Value
**Upregulated genes**				
1371193_at	Tnfaip6	tumor necrosis factor alpha induced protein 6	2.91	0.02394
1379200_at	RGD1563278	DAZ interacting zinc finger protein 3	2.49	0.00296
1369692_at	Tnr	tenascin R	2.08	0.01208
1374730_at	Tyrobp	tyro protein tyrosine kinase binding protein	2.07	0.03243
1369756_a_at	Slc4a5	solute carrier family 4, sodium bicarbonate cotransporter, member 5	1.99	0.04417
1371250_at	Pf4	platelet factor 4	1.92	0.00927
1376823_at	Fndc3a	fibronectin type III domain containing 3a	1.91	0.04421
1370946_at	Nfix	nuclear factor I/X (CCAAT-binding transcription factor)	1.89	0.04099
1379397_at	Rora	RAR-related orphan receptor A	1.88	0.00928
1367934_at	Rpl39	ribosomal protein L39	1.76	0.02017
1388185_at	LOC683869	similar to Retinoblastoma-like protein 1 (107 kDa retinoblastoma-associated protein) (PRB1) (P107)	1.71	0.04993
1385168_at	RGD1306520	similar to receptor-interacting factor 1	1.53	0.04513
1385751_at	Thbs2	thrombospondin 2	1.48	0.01937
1372404_at	Rac2	ras-related C3 botulinum toxin substrate 2 (rho family, small GTP binding protein Rac2)	1.46	0.00753
1386869_at	LOC360902	similar to actin, gamma 2, smooth muscle, enteric	1.45	0.01126
1394125_at	RGD1563410	similar to put. type 5 nonmuscle actin	1.45	0.01126
1371447_at	Plac8	placenta-specific 8	1.45	0.02442
1383911_at	Kdm4c	lysine (K)-specific demethylase 4C	1.44	0.02333
1371106_at	Itgb8	integrin, beta 8	1.43	0.02808
1390938_at	Arhgap30	rho GTPase activating protein 30	1.42	0.00963
1373884_at	Klhl2	kelch-like 2, Mayven (Drosophila)	1.41	0.03500
1373504_at	LOC679819	GLI pathogenesis-related 2	1.41	0.02410
1368482_at	Bcl2a1d	b-cell leukemia/lymphoma 2 related protein A1d	1.36	0.04885
1371015_at	Mx1	myxovirus (influenza virus) resistance 1	1.36	0.00048
1383410_at	Srp54a	signal recognition particle 54a	1.34	0.01999
1384320_at	Bai3	brain-specific angiogenesis inhibitor 3	1.31	0.04855
1370767_at	Cox6c1	cytochrome c oxidase subunit VIc-1	1.31	0.00696
1390782_at	Clec14a	C-type lectin domain family 14, member a	1.31	0.04408
1393863_at	Cd180	cd180 molecule	1.30	0.01662
1373195_at	Fus	fusion (involved in t(12;16) in malignant liposarcoma) (human)	1.28	0.01418
1374734_at	LOC680254	hypothetical protein LOC680254	1.23	0.03038
1388484_at	Ube2c	ubiquitin-conjugating enzyme E2C	1.21	0.02231
1390510_at	Ms4a6b	membrane-spanning 4-domains, subfamily A, member 6B	1.18	0.02870
1391754_at	Oas1i	2 ' -5 ' oligoadenylate synthetase 1I	1.17	0.03340
1371859_at	Poldip3	polymerase (DNA-directed), delta interacting protein 3	1.16	0.00218
1381757_at	RGD1309501	hypothetical LOC305552	1.15	0.03231
1368418_a_at	Cp	ceruloplasmin	1.15	0.00244
1381758_at	RGD1565672	similar to MDM2 Binding protein	1.12	0.01732
1377642_at	Cav2	caveolin 2	1.12	0.04826
1396803_at	Thoc2	THO complex 2	1.09	0.03370
1396239_at	RGD1566036	similar to RIKEN cDNA 2310008H04	1.09	0.01726
1385434_at	Ptger2	prostaglandin E receptor 2 (subtype EP2)	1.08	0.00560
1386913_at	Pdpn	podoplanin	1.07	0.01952
1394144_at	Tubb6	tubulin, beta 6	1.06	0.00314
1387995_a_at	Ifitm3	interferon induced transmembrane protein 3	1.06	0.00281
1372703_at	LOC689226	similar to ubiquitin-conjugating enzyme E2R 2	1.05	0.01019
1383747_at	Ect2	epithelial cell transforming sequence 2 oncogene	1.05	0.03814
1382913_at	Cttnbp2	cortactin binding protein 2	1.04	0.00510
1379287_at	RGD1565766	hypothetical gene supported by BC088468; NM_001009712	1.04	0.04919
1373025_at	C1qc	complement component 1, q subcomponent, C chain	1.03	0.00114
1398279_at	Trpv5	transient receptor potential cation channel, subfamily V, member 5	1.02	0.00499
1396918_at	LOC362852	similar to ubiquitin conjugating enzyme E2	1.00	0.00543
1378914_a_at	LOC308990	hypothetical protein LOC308990	1.00	0.01307
1370977_at	Dpf1	D4, zinc and double PHD fingers family 1	0.99	0.01834
1371016_at	LOC290071	similar to RIKEN cDNA A430107P09 gene-like /// similar to RIKEN cDNA A430107P09 gene	0.99	0.00683
1394678_at	Fgd2	FYVE, RhoGEF and PH domain containing 2	0.98	0.04659
1373490_at	Gmfg	glia maturation factor, gamma	0.96	0.03901
1388906_at	RGD1564930	predicted similar to novel protein similar to Tensin Tns (predicted)	0.95	0.04029
1373401_at	Tnc	tenascin C	0.94	0.01809
1393110_at	Mpv17l	MPV17 mitochondrial membrane protein-like	0.93	0.01710
1394744_at	Eef1g	Eukaryotic translation elongation factor 1 gamma	0.93	0.00067
1378338_at	RGD1561004	alport syndrome, mental retardation, midface hypoplasia and elliptocytosis chromosomal region gene 1	0.93	0.03967
1383863_at	Lmo2	LIM domain only 2	0.92	0.02360
1383320_at	Lck	lymphocyte-specific protein tyrosine kinase	0.92	0.01487
1381533_at	Rnd1	rho family GTPase 1	0.91	0.04627
1368186_a_at	Syk	spleen tyrosine kinase	0.91	0.01985
1371660_at	LOC682099	similar to Juxtaposed with another zinc finger protein 1	0.91	0.00208
1371162_at	Mrga10	MAS-related GPR, member A10	0.90	0.01465
1375718_at	LOC498525	bm403207	0.89	0.00824
1376704_a_at	Ndnl2	necdin-like 2	0.89	0.02869
1383616_at	Il10rb	interleukin 10 receptor, beta	0.89	0.00712
1385724_at	LOC680752	coiled-coil domain containing 74A	0.89	0.04562
1392703_at	Tbx4	T-box 4	0.88	0.04633
1372725_at	LOC315883	phospholipid scramblase 2	0.87	0.03304
1372827_at	RGD1560149	peptidylprolyl isomerase D-like 1	0.87	0.01169
1372641_at	Alpk3	alpha-kinase 3	0.86	0.03662
1372809_at	LOC290595	hypothetical gene supported by AF152002	0.86	0.00726
1376974_at	Ttc7	tetratricopeptide repeat domain 7	0.85	0.00438

Probe ID	Gene symbol	Gene full name	Log2 (fold change)	*p* Value
1375847_at	Nudt16	nudix (nucleoside diphosphate linked moiety X)-type motif 16	0.85	0.03311
1377080_at	Nradd	neurotrophin receptor associated death domain	0.84	0.03976
1376447_at	RGD1565772	similar to hypothetical protein A430110N23	0.84	0.01177
1383925_at	LOC298643	migration and invasion inhibitory protein	0.84	0.00212
1390641_at	Zfp346	zinc finger protein 346	0.83	0.01757
1385627_at	LOC686871	similar to B-cell leukemia/lymphoma 3	0.81	0.02848
1385627_at	LOC680611	bcl3 B-cell CLL/lymphoma 3	0.81	0.02848
1367574_at	Vim	vimentin	0.81	0.03826
1379315_at	Rassf7	ras association (RalGDS/AF-6) domain family (N-terminal) member 7	0.81	0.00943
1393510_at	Golsyn	golgi-localized protein	0.81	0.00159
1399104_at	Crebbp	CREB binding protein	0.81	0.01083
1383647_a_at	Crb3	crumbs homolog 3 (Drosophila)	0.80	0.04472
1392662_at	Cisd2	CDGSH iron sulfur domain 2	0.80	0.04512
1396478_at	Tmem164	transmembrane protein 164	0.79	0.04547
1371777_at	Pabpc4	poly(A) binding protein, cytoplasmic 4	0.79	0.00736
1375741_at	Ccdc88b	coiled-coil domain containing 88B	0.79	0.00635
1384528_at	Rai14	retinoic acid induced 14	0.79	0.02043
1367844_at	Gnai2	guanine nucleotide binding protein (G protein), alpha inhibiting 2	0.79	0.03517
1372672_at	Qprt	quinolinate phosphoribosyltransferase	0.78	0.00045
1392466_at	Tmem135	transmembrane protein 135	0.77	0.03609
1393460_at	Lrrc33	leucine rich repeat containing 33	0.77	0.03432
1377848_at	Ccdc98	coiled-coil domain containing 98	0.76	0.03590
1370913_at	Rsad2	radical S-adenosyl methionine domain containing 2	0.76	0.01576
1380262_at	Sgk2	serum/glucocorticoid regulated kinase 2	0.76	0.04691
1368429_at	Taf9b	TAF9B RNA polymerase II, TATA box binding protein (TBP)-associated factor	0.75	0.03417
1369841_at	Hspa2	heat shock protein alpha 2	0.75	0.01776
1395404_at	Fn3krp	fructosamine-3-kinase-related protein	0.75	0.04185
1371074_a_at	Mcm6	minichromosome maintenance complex component 6	0.74	0.04006
1375633_at	Clic1	chloride intracellular channel 1	0.73	0.03463
1398250_at	Acot1	acyl-CoA thioesterase 1	0.73	0.04288
1374204_at	Wsb1	WD repeat and SOCS box-containing 1	0.73	0.04142
1382315_at	Tsc22d4	TSC22 domain family, member 4	0.73	0.00227
1370892_at	C4b	complement component 4, gene 2 /// complement component 4B (Chido blood group)	0.72	0.04977
1389686_at	Prkx	protein kinase, X-linked	0.72	0.00888
1389828_at	Cebpd	CCAAT/enhancer binding protein (C/EBP), delta	0.72	0.01852
1374540_at	Cdca7	cell division cycle associated 7	0.72	0.00494
1379853_at	Hspb11	heat shock protein family B (small), member 11	0.71	0.00684
1389364_at	Ndfip2	Nedd4 family interacting protein 2	0.71	0.04793
1371550_at	LOC686226	similar to TSC22 domain family protein 4 (TSC22-related-inducible leucine zipper protein 2)	0.71	0.03512
1368308_at	Myc	myelocytomatosis oncogene	0.70	0.02386
1389271_at	LOC679921	vasorin	0.70	0.01754
1369161_at	Abcb4	ATP-binding cassette, sub-family B (MDR/TAP), member 4	0.70	0.00562
1382364_at	RGD1310427	similar to KIAA0090 protein	0.70	0.00050
1390088_at	Nog	noggin	0.70	0.04369
1379914_at	Klf11	kruppel-like factor 11	0.70	0.02425
1367549_a_at	Ap3d1	adaptor-related protein complex 3, delta 1 subunit	0.70	0.00450
1383940_at	Nuf2	NUF2, NDC80 kinetochore complex component, homolog (S. cerevisiae)	0.69	0.03343
1385430_at	Lims1	LIM and senescent cell antigen-like domains 1	0.68	0.04999
1374401_at	Snx2	sorting nexin 2	0.67	0.01603
1378353_at	Setdb1	SET domain, bifurcated 1	0.67	0.00473
1390287_at	Bin2	bridging integrator 2	0.67	0.01688
1384714_x_at	RGD1561333	similar to 60S ribosomal protein L8 /// ribosomal protein L8	0.67	0.04153
1367800_at	Plat	plasminogen activator, tissue	0.66	0.00192
1368970_at	Cdh23	cadherin 23 (otocadherin)	0.66	0.00395
1378127_at	Cul2	cullin 2	0.66	0.01152
1371411_at	Plxnb2	plexin B2	0.66	0.03683
1371758_at	Ufc1	ubiquitin-fold modifier conjugating enzyme 1	0.66	0.04901
1384099_at	Slc39a6	solute carrier family 39 (zinc transporter), member 6	0.66	0.01167
1393724_at	LOC362056	spermatogenesis associated 1	0.66	0.00142
1372640_at	Pi16	peptidase inhibitor 16	0.66	0.01815
1388755_at	Sec23a	sec23 homolog A (S. cerevisiae)	0.65	0.04085
1368259_at	Ptgs1	prostaglandin-endoperoxide synthase 1	0.65	0.02145
1370857_at	Acta2	smooth muscle alpha-actin	0.65	0.03860
1372548_at	Cry2	cryptochrome 2 (photolyase-like)	0.65	0.00061
1373623_at	Itpkc	inositol 1,4,5-trisphosphate 3-kinase C	0.65	0.02807
1372254_at	Serping1	serine (or cysteine) peptidase inhibitor, clade G, member 1	0.65	0.03990
1374055_at	Erf	ets2 repressor factor	0.65	0.02556
1375436_at	Naprt1	nicotinate phosphoribosyltransferase domain containing 1	0.64	0.00192
1379458_at	Klhl14	kelch-like 14 (Drosophila)	0.64	0.03486
1367871_at	Cyp2e1	cytochrome P450, family 2, subfamily e, polypeptide 1	0.64	0.01934
1385333_at	LOC688299	similar to apoptosis-associated tyrosine kinase	0.64	0.01980
1385333_at	LOC690853	apoptosis-associated tyrosine kinase	0.64	0.01980
1373625_at	Shmt1	serine hydroxymethyltransferase 1 (soluble)	0.64	0.01600
1388496_at	Flnc	filamin C, gamma	0.64	0.02826
1395311_at	Icmt	isoprenylcysteine carboxyl methyltransferase	0.64	0.01974
1368148_at	Ngfr	nerve growth factor receptor (TNFR superfamily, member 16)	0.62	0.01037
1367832_at	Lypla1	lysophospholipase 1	0.62	0.04981
1369511_at	Ednra	endothelin receptor type A	0.61	0.02946
1382104_at	Wdr33	WD repeat domain 33	0.61	0.00060
1390933_a_at	Rg9mtd3	RNA (guanine-9-) methyltransferase domain containing 3	0.61	0.00022
1371499_at	Cd9	cd9 molecule	0.61	0.00179

Probe ID	Gene symbol	Gene full name	Log2 (fold change)	*p* Value
1374537_at	Chsy1	chondroitin sulfate synthase 1	0.60	0.01798
1387546_at	LOC502603	serine/arginine-rich splicing factor 11	0.60	0.00186
1377626_at	LOC690768	hypothetical protein LOC690768	0.59	0.00657
1387272_at	Alpk1	alpha-kinase 1	0.59	0.02031
1387889_at	Folr1	folate receptor 1 (adult)	0.59	0.00247
1374452_at	Pde9a	phosphodiesterase 9A	0.59	0.01891
1377984_at	Itpkb	inositol 1,4,5-trisphosphate 3-kinase B	0.59	0.03313
1383671_at	Adam9	ADAM metallopeptidase domain 9 (meltrin gamma)	0.59	0.03313
**Downregulated genes**				
1394921_at	Prkaa1	protein kinase, AMP-activated, alpha 1 catalytic subunit	-2.00	0.01959
1384971_at	Depdc6	DEP domain containing 6	-1.98	0.00285
1368116_a_at	Rps6kb1	ribosomal protein S6 kinase, 70kDa, polypeptide 1	-1.77	0.01475
1377061_at	Rics	rho GTPase-activating protein	-1.59	0.00107
1392133_at	Mapk1ip1l	mitogen-activated protein kinase 1 interacting protein 1-like	-1.52	0.04554
1385017_at	LOC691610	zinc finger protein 770	-1.51	0.00359
1369860_a_at	Htr2c	5-hydroxytryptamine (serotonin) receptor 2C	-1.47	0.03263
1369248_a_at	Xiap	X-linked inhibitor of apoptosis	-1.36	0.02584
1387374_at	Tcf12	transcription factor 12	-1.34	0.02482
1387716_at	Utrn	utrophin	-1.34	0.03705
1369540_at	Necab1	N-terminal EF-hand calcium binding protein 1	-1.33	0.01087
1393910_at	Fam13a1	family with sequence similarity 13, member A1	-1.33	0.02288
1388218_at	Ldlr	low density lipoprotein receptor	-1.31	0.03026
1387737_at	Mat2a	methionine adenosyltransferase II, alpha	-1.26	0.04118
1394049_at	Dcun1d1	DCN1, defective in cullin neddylation 1, domain containing 1 (S. cerevisiae)	-1.22	0.00848
1369903_at	Gabrb3	gamma-aminobutyric acid (GABA) A receptor, beta 3	-1.21	0.00482
1367728_at	Tsn	translin	-1.21	0.02869
1372103_at	Dnaja4	dnaJ (Hsp40) homolog, subfamily A, member 4	-1.21	0.02946
1393136_at	LOC313618	transmembrane protein 57	-1.19	0.03175
1384797_at	Atl3	atlastin GTPase 3	-1.18	0.03678
1369332_a_at	Rims1	regulating synaptic membrane exocytosis 1	-1.16	0.02805
1370991_at	Cml3	camello-like 3	-1.15	0.02183
1388748_at	Laptm4a	lysosomal protein transmembrane 4 alpha	-1.14	0.00000
1371706_at	LOC683605	similar to serologically defined colon cancer antigen 3 isoform 1	-1.13	0.05000
1384106_at	Sgms1	sphingomyelin synthase 1	-1.12	0.00812
1369421_at	Top1	topoisomerase (DNA) I	-1.12	0.00933
1395600_at	LOC498404	coiled-coil domain containing 117	-1.12	0.04388
1388171_at	Cdk7	cyclin-dependent kinase 7	-1.10	0.00038
1390179_at	Ankrd52	ankyrin repeat domain 52	-1.09	0.03991
1379248_at	Prcp	prolylcarboxypeptidase (angiotensinase C)	-1.08	0.00584
1370833_at	Pex5l	peroxisomal biogenesis factor 5-like	-1.08	0.01238
1382306_at	Arih1	ariadne ubiquitin-conjugating enzyme E2 binding protein homolog 1 (Drosophila)	-1.07	0.00268
1387929_at	Pmf31	PMF32 protein	-1.07	0.01813
1398458_at	RGD1307284	lysine deficient protein kinase 2	-1.04	0.00003
1395344_at	Mtmr1	myotubularin related protein 1	-1.03	0.02156
1393306_at	Cul5	Cullin 5	-1.02	0.00363
1387824_at	Sfrs12	splicing factor, arginine/serine-rich 12	-1.01	0.00274
1377643_at	Hoxd10	homeo box D10	-1.01	0.02313
1387301_at	Fgf1	fibroblast growth factor 1	-1.01	0.04044
1393016_s_at	Sec3l1	SEC3-like 1 (S. cerevisiae)	-1.00	0.01256
1387137_at	Comp	cartilage oligomeric matrix protein	-0.99	0.04265
1369265_at	Senp2	Sumo1/sentrin/SMT3 specific peptidase 2	-0.97	0.04040
1370116_at	Sept3	septin 3	-0.97	0.01051
1386023_at	Lgi1	leucine-rich, glioma inactivated 1 /// leucine-rich glioma-inactivated protein 1-like	-0.96	0.00570
1370520_at	LOC257650	hippyragranin	-0.96	0.01273
1387204_at	Negr1	neuronal growth regulator 1	-0.95	0.02301
1369044_a_at	Pde4b	phosphodiesterase 4B, cAMP specific	-0.95	0.01430
1387529_a_at	Tagln3	transgelin 3	-0.95	0.00004
1368889_at	Vti1a	vesicle transport through interaction with t-SNAREs homolog 1A (yeast)	-0.95	0.03709
1379778_at	DMXL1	Dmx-like 1	-0.95	0.04442
1375627_at	Akirin2	akirin 2	-0.94	0.04917
1382171_at	LOC499624	TSC22 domain family, member 2	-0.94	0.03081
1376195_at	Spin1	spindlin 1	-0.94	0.02230
1396131_at	Fam134c	family with sequence similarity 134, member C	-0.94	0.01194
1368573_at	Kpnb1	karyopherin (importin) beta 1	-0.93	0.04213
1386685_at	Sacs	spastic ataxia of Charlevoix-Saguenay (sacsin)	-0.92	0.01311
1388022_a_at	Dnm1l	dynamin 1-like	-0.92	0.00877
1368438_at	Pde10a	phosphodiesterase 10A	-0.92	0.03902
1367996_a_at	Lphn1	latrophilin 1	-0.91	0.00629
1377025_at	Kif3a	kinesin family member 3a	-0.89	0.04134
1375015_at	LOC317546	similar to TAK1-binding protein 3 isoform 1	-0.89	0.04637
1387807_at	Pafah1b1	platelet-activating factor acetylhydrolase, isoform 1b, subunit 1	-0.88	0.00180
1370016_at	Nell2	NEL-like 2 (chicken)	-0.88	0.04451
1381098_at	LOC690243	hypothetical protein LOC690243	-0.88	0.01710
1372977_at	Map4k5	mitogen-activated protein kinase kinase kinase kinase 5	-0.88	0.03309
1378096_at	Kif5b	kinesin family member 5B	-0.87	0.02917
1369041_at	Nlgn1	neuroligin 1	-0.87	0.02291
1371800_at	Phc3	polyhomeotic homolog 2 (Drosophila)	-0.86	0.03311
1388143_at	Col18a1	collagen, type XVIII, alpha 1	-0.86	0.00323
1375978_at	Fcho1	FCH domain only 1	-0.85	0.02250
1385835_at	Plxdc1	plexin domain containing 1	-0.85	0.00177
1373913_at	Pnpt1	polyribonucleotide nucleotidyltransferase 1	-0.84	0.01486

Probe ID	Gene symbol	Gene full name	Log2 (fold change)	*p* Value
1375616_at	Apba2	Amyloid beta (A4) precursor protein-binding, family A, member 2	-0.83	0.03022
1375226_at	Tbc1d10b	TBC1 domain family, member 10b	-0.83	0.01106
1368369_at	Pnoc	prepronociceptin	-0.83	0.00758
1369720_at	Myo1b	myosin Ib	-0.82	0.02531
1382451_at	Hebp2	heme binding protein 2	-0.82	0.01222
1397824_at	RGD1562407	similar to WAC	-0.82	0.03809
1384002_at	LOC361929	ATPase, class VI, type 11B	-0.82	0.03022
1386897_at	LOC500378	chromatin target of PRMT1	-0.81	0.03586
1374521_at	Pcgf3	polycomb group ring finger 3	-0.80	0.00238
1395850_at	Pus3	pseudouridylate synthase 3	-0.80	0.03211
1379612_at	Mapk8	mitogen-activated protein kinase 8	-0.80	0.02826
1384205_at	Ngly1	N-glycanase 1	-0.80	0.00304
1389032_at	Mcoln1	mucolipin 1	-0.80	0.01838
1388274_at	Bmyc	brain expressed myelocytomatosis oncogene	-0.79	0.04070
1391016_at	LOC690654	tryptophanyl tRNA synthetase 2 (mitochondrial)	-0.79	0.00324
1391619_at	Bhlhb9	basic helix-loop-helix domain containing, class B, 9	-0.79	0.03423
1387224_at	Dgkb	diacylglycerol kinase, beta	-0.79	0.01798
1369693_a_at	Slc1a2	solute carrier family 1 (glial high affinity glutamate transporter), member 2	-0.79	0.04256
1370081_a_at	Vegfa	vascular endothelial growth factor A	-0.78	0.02115
1384745_at	Piwil2	piwi-like 2 (Drosophila)	-0.78	0.02224
1390455_at	Abhd2	abhydrolase domain containing 2	-0.78	0.04928
1369024_at	Rabep2	rabaptin, RAB GTPase binding effector protein 2	-0.78	0.01616
1375545_at	Rbm9	RNA binding motif protein 9	-0.78	0.00509
1382366_at	Pik3cb	phosphoinositide-3-kinase, catalytic, beta polypeptide	-0.78	0.04318
1368615_a_at	Slc18a3	solute carrier family 18 (vesicular acetylcholine), member 3	-0.78	0.02197
1369687_at	Kcnab3	potassium voltage-gated channel, shaker-related subfamily, beta member 3	-0.76	0.03811
1369820_at	Mcf2l	MCF.2 cell line derived transforming sequence-like	-0.76	0.02718
1377750_at	Arhgef3	rho guanine nucleotide exchange factor (GEF) 3	-0.75	0.01941
1377806_at	Vezf1	vascular endothelial zinc finger 1	-0.75	0.00094
1387837_at	Apc	adenomatous polyposis coli	-0.75	0.03805
1393555_at	Hsp90ab1	heat shock protein 90kDa alpha (cytosolic), class B member 1	-0.75	0.00067
1388738_at	Atrnl1	Attractin like 1	-0.74	0.04906
1367656_at	Psmb7	proteasome (prosome, macropain) subunit, beta type 7	-0.74	0.03573
1375245_at	LOC308398	protein phosphatase 1, regulatory subunit 37	-0.73	0.00797
1394923_at	LOC683559	similar to nuclear receptor interacting protein 2	-0.73	0.01139
1395988_at	Zdhhc22	zinc finger, DHHC-type containing 22	-0.73	0.00837
1373993_at	LOC686858	similar to small nuclear RNA activating complex, polypeptide 5	-0.73	0.03192
1393236_at	Riok3	RIO kinase 3 (yeast)	-0.73	0.00947
1387563_at	Pgr	progesterone receptor	-0.73	0.01073
1391693_at	Atp8a1	ATPase, Class I, type 8A, member 1	-0.72	0.00587
1379558_at	LOC680222	similar to zinc finger protein 748 isoform 2	-0.71	0.00211
1389646_at	Cdc23	cdc23 (cell division cycle 23, yeast, homolog)	-0.71	0.01061
1396919_at	Wnk1	WNK lysine deficient protein kinase 1	-0.71	0.00531
1371317_at	LMO1	LIM domain only 1	-0.70	0.04124
1377288_at	Hsf4	heat shock transcription factor 4	-0.70	0.04045
1374637_at	Tmco6	transmembrane and coiled-coil domains 6	-0.70	0.02520
1383326_a_at	Pdcd4	programmed cell death 4	-0.70	0.04120
1377582_at	Tox3	TOX high mobility group box family member 3	-0.70	0.03290
1384879_at	Trpt1	tRNA phosphotransferase 1	-0.70	0.03907
1386253_at	Med4	mediator complex subunit 4	-0.69	0.00226
1373045_at	Kctd13	potassium channel tetramerisation domain containing 13	-0.69	0.02235
1368177_at	Acsl3	acyl-CoA synthetase long-chain family member 3	-0.69	0.03584
1367796_at	Mgat1	mannosyl (alpha-1,3-)-glycoprotein beta-1,2-N-acetylglucosaminyltransferase	-0.69	0.00830
1375305_at	RGD1563912	small nucleolar RNA host gene 11 (non-protein coding)	-0.69	0.00858
1374642_at	Zfp64	zinc finger protein 64	-0.68	0.04765
1388750_at	Tfrc	transferrin receptor	-0.68	0.00065
1390976_at	Lynx1	Ly6/neurotoxin 1	-0.68	0.01879
1387435_at	St8sia3	ST8 alpha-N-acetyl-neuraminide alpha-2,8-sialyltransferase 3	-0.68	0.01910
1392755_at	Lrrc8c	leucine rich repeat containing 8 family, member C	-0.68	0.03399
1370757_at	Cacng3	calcium channel, voltage-dependent, gamma subunit 3	-0.68	0.02102
1393363_at	LOC305913	zinc finger, MYM-type 2	-0.68	0.02175
1368421_at	Ptpn5	protein tyrosine phosphatase, non-receptor type 5	-0.67	0.01549
1370112_at	Pten	phosphatase and tensin homolog	-0.67	0.01603
1369680_at	Slc2a13	solute carrier family 2 (facilitated glucose transporter), member 13	-0.67	0.03371
1389865_at	Toe1	target of EGR1, member 1 (nuclear)	-0.67	0.01512
1373542_at	Sphk2	sphingosine kinase 2	-0.66	0.03857
1388761_at	Hdac1	histone deacetylase 1	-0.66	0.01774
1389037_at	Rit1	Ras-like without CAAX 1	-0.66	0.04310
1370535_at	Myt1l	myelin transcription factor 1-like	-0.66	0.01021
1383608_at	Arrdc2	arrestin domain containing 2	-0.66	0.01961
1368026_at	Hdgfrp2	hepatoma-derived growth factor, related protein 2	-0.66	0.00168
1385229_at	Pcdh20	protocadherin 20	-0.66	0.02724
1377696_at	Mest	mesoderm specific transcript homolog (mouse)	-0.66	0.01578
1372506_at	Psme3	proteasome (prosome, macropain) activator subunit 3	-0.66	0.00616
1373061_at	Snx17	sorting nexin 17	-0.65	0.02691
1386279_at	Stard4	StAR-related lipid transfer (START) domain containing 4	-0.65	0.01282
1387873_at	Wfdc1	WAP four-disulfide core domain 1	-0.65	0.00182
1368082_at	Slc4a2	solute carrier family 4 (anion exchanger), member 2	-0.65	0.02171
1370824_at	Slc38a3	solute carrier family 38, member 3	-0.65	0.00507
1374568_at	RGD1309492	similar to mKIAA1737 protein	-0.65	0.03285
1386918_a_at	Sigmar1	sigma non-opioid intracellular receptor 1	-0.65	0.00329

Probe ID			Log2 (fold change)	*p* Value
1373075_at	RGD1560888	similar to Cell division protein kinase 8 (Protein kinase K35)	-0.65	0.00411
1387103_s_at	Slc25a14	solute carrier family 25 (mitochondrial carrier, brain), member 14	-0.65	0.04811
1368621_at	Aqp9	aquaporin 9	-0.64	0.02930
1369269_at	Galnt1	UDP-N-acetyl-alpha-D-galactosamine:polypeptide N-acetylgalactosaminyltransferase 1 (GalNAc-T1)	-0.64	0.01425
1389167_at	Mapkap1	mitogen-activated protein kinase associated protein 1	-0.64	0.00520
1385218_at	Noc3l	nucleolar complex associated 3 homolog (S. cerevisiae)	-0.64	0.04356
1370100_at	Pik3r2	phosphoinositide-3-kinase, regulatory subunit 2 (beta)	-0.64	0.02661
1371060_at	Trim23	tripartite motif-containing 23	-0.64	0.04303
1371126_x_at	RGD1305094	similar to CG2662-PA	-0.63	0.02234
1368922_at	Ecel1	endothelin converting enzyme-like 1	-0.63	0.04031
1374470_at	Dhx57	DEAH (Asp-Glu-Ala-Asp/His) box polypeptide 57	-0.63	0.01705
1374640_at	Them4	thioesterase superfamily member 4	-0.63	0.00645
1376192_at	Nat9	N-acetyltransferase 9 (GCN5-related, putative)	-0.63	0.00547
1372527_at	Rtn2	reticulon 2	-0.63	0.01577
1372900_at	Pigt	phosphatidylinositol glycan anchor biosynthesis, class T	-0.63	0.03633
1379309_at	Tbl1xr1	transducin (beta)-like 1 X-linked receptor 1	-0.63	0.02315
1388503_at	Eid1	EP300 interacting inhibitor of differentiation 1	-0.63	0.04234
1388953_at	Gnl3	guanine nucleotide binding protein-like 3 (nucleolar)	-0.63	0.00951
1374626_at	Lrg1	leucine-rich alpha-2-glycoprotein 1	-0.63	0.04426
1376408_at	LOC499339	hypothetical protein LOC499339	-0.62	0.00701
1384903_at	Gpt2	glutamic pyruvate transaminase (alanine aminotransferase) 2	-0.62	0.03375
1378506_at	Pik3c2a	phosphoinositide-3-kinase, class 2, alpha polypeptide	-0.62	0.04098
1382386_at	Lmbr1	limb region 1 homolog (mouse)	-0.62	0.00078
1368439_at	Sox10	SRY (sex determining region Y)-box 10	-0.62	0.03760
1383867_at	Eif5a2	eukaryotic translation initiation factor 5A2	-0.62	0.00750
1372536_at	Cabc1	chaperone, ABC1 activity of bc1 complex homolog (S. pombe)	-0.62	0.02433
1370490_at	Pcdhb12	protocadherin beta 12	-0.62	0.01867
1395464_at	RGD1311154	potassium channel tetramerization domain containing 17	-0.62	0.00367
1388697_at	Inpp5a	inositol polyphosphate-5-phosphatase A	-0.62	0.01424
1393565_at	RGD1563441	similar to RIKEN cDNA A030009H04	-0.61	0.04674
1396462_at	Vps4a	vacuolar protein sorting 4 homolog A (S. cerevisiae)	-0.61	0.03118
1374323_at	Bccip	BRCA2 and CDKN1A interacting protein	-0.61	0.01895
1373498_at	Vps11	vacuolar protein sorting 11 homolog (S. cerevisiae)	-0.61	0.02517
1389377_at	Insig2	insulin induced gene 2	-0.61	0.01780
1395066_at	Nol4	nucleolar protein 4	-0.61	0.04471
1367980_at	Rabep1	rabaptin, RAB GTPase binding effector protein 1	-0.61	0.04047
1370954_at	P4ha1	prolyl 4-hydroxylase, alpha polypeptide I	-0.60	0.02564
1388019_at	Odf2	outer dense fiber of sperm tails 2	-0.60	0.03901
1396052_at	Dhx8	DEAH (Asp-Glu-Ala-His) box polypeptide 8	-0.60	0.00391
1375398_at	Tmem163	transmembrane protein 163	-0.60	0.00767
1384445_at	LOC298442	coiled-coil domain containing 163	-0.60	0.02437
1376607_a_at	RGD1310414	similar to hypothetical protein FLJ23263	-0.60	0.03785
1382251_at	Kpna1	karyopherin alpha 1	-0.60	0.04425
1381279_at	Ripk2	receptor-interacting serine-threonine kinase 2	-0.60	0.01095
1387801_at	Ppp6c	protein phosphatase 6, catalytic subunit	-0.59	0.03892
1369562_at	Hpcal1	hippocalcin-like 1	-0.59	0.03476
1372228_at	Asmtl	acetylserotonin O-methyltransferase-like	-0.59	0.00921
1369213_at	L1cam	L1 cell adhesion molecule	-0.59	0.03084
1378116_at	Zbtb45	zinc finger and BTB domain containing 45	-0.59	0.03166
1384423_at	LOC365090	similar to 5-nucleotidase, cytosolic II	-0.59	0.03230
1374152_at	Wdr46	WD repeat domain 46	-0.59	0.03593
1371585_at	Gspt1	G1 to S phase transition 1	-0.59	0.04259
1387979_at	Golgb1	golgi autoantigen, golgin subfamily b, macrogolgin 1	-0.59	0.00603
1390945_at	Znf292	zinc finger protein 292	-0.59	0.02462
