# Supplementary material for: Molecular and Cellular Changes in the Lumbar Spinal Cord following Thoracic Injury: Regulation by Treadmill Locomotor Training
Source: PLoS One. 2014 Feb 10;9(2):e88215. doi: 10.1371/journal.pone.0088215 (PMC3919755; doi:10.1371/journal.pone.0088215)
Supplement: Table S3 — A list of genes that were differentially expressed only at 3-week time point. (PDF) [file pone.0088215.s003.pdf]

Table S3. A list of genes that were differentially expressed at both 1- and 3-week time point

Probe ID	Gene symbol	Gene full name	1W Log2 (fold change)	*p* Value	3W Log2 (fold change)	*p* Value
		**Upregulated genes**				
1386879_at	Lgals3	lectin, galactoside-binding, soluble, 3	2.53	0.01401	3.40	0.00076
1368000_at	C3	complement component 3	2.25	0.02035	1.64	0.01661
1393682_at	Gpr34	G protein-coupled receptor 34	2.24	0.01051	2.57	0.00230
1375010_at	Cd68	cd68 molecule	2.20	0.04382	2.61	0.01059
1374367_at	Grxcr1	glutaredoxin, cysteine rich 1	2.17	0.00856	1.84	0.00606
1368558_s_at	Aif1	allograft inflammatory factor 1	2.13	0.01066	1.73	0.02598
1368187_at	Gpnmb	glycoprotein (transmembrane) nmb	2.13	0.03007	3.56	0.00090
1367712_at	Timp1	TIMP metallopeptidase inhibitor 1	2.11	0.01815	1.87	0.00398
1393280_at	Ly86	lymphocyte antigen 86	2.03	0.00518	1.34	0.00534
1390687_at	Plek	pleckstrin	1.84	0.00961	1.70	0.00636
1368382_at	S100a3	s100 calcium binding protein A3	1.76	0.02629	1.84	0.03418
1389553_at	Clec4a3	C-type lectin domain family 4, member a3	1.75	0.00867	1.39	0.04559
1387029_at	Cfh	complement factor H	1.74	0.00014	1.88	0.02218
1388271_at	LOC689415	metallothionein 2A	1.74	0.03042	1.88	0.00091
1390798_at	Ptprc	protein tyrosine phosphatase, receptor type, C	1.73	0.01557	1.14	0.03096
1386936_at	Grifin	galectin-related inter-fiber protein	1.73	0.00853	1.94	0.02674
1393641_at	Blnk	b-cell linker	1.72	0.04888	1.60	0.00180
1373412_at	LOC295635	similar to 5-nucleotidase, cytosolic II	1.68	0.02667	1.13	0.03131
1378418_at	Tifab	TRAF-interacting protein with forkhead-associated domain, family member B	1.66	0.01154	1.16	0.03754
1386009_at	Csf3r	colony stimulating factor 3 receptor (granulocyte)	1.63	0.00609	1.30	0.00327
1373504_at	Glipr1	GLI pathogenesis-related 1	1.61	0.02282	1.54	0.00155
1388689_at	LOC682245	similar to Acylphosphatase, muscle type isozyme (Acylphosphate phosphohydrolase)	1.61	0.02475	2.61	0.03861
1370080_at	Hmox1	heme oxygenase (decycling) 1	1.59	0.04623	1.06	0.02284
1379766_at	Sla	Src-like adaptor	1.53	0.03350	1.20	0.00452
1377153_a_at	Klhl6	kelch-like 6 (Drosophila)	1.53	0.01662	1.05	0.01206
1394673_at	LOC687856	similar to Myeloid cell surface antigen CD33 precursor (Siglec-3)	1.51	0.01933	1.60	0.00821
1368270_at	Apobec1	apolipoprotein B mRNA editing enzyme, catalytic polypeptide 1	1.50	0.01534	1.58	0.01536
1370249_at	Tspo	translocator protein	1.49	0.01378	1.35	0.00348
1378140_at	Arl11	ADP-ribosylation factor-like 11	1.48	0.02064	1.94	0.00343
1381311_at	Emr1	EGF-like module containing, mucin-like, hormone receptor-like 1	1.48	0.01314	1.88	0.01253
1372397_at	LOC686733	similar to TIP41, TOR signalling pathway regulator-like	1.45	0.03628	1.91	0.04804
1372397_at	Tiprl	TIP41, TOR signaling pathway regulator-like (S. cerevisiae)	1.45	0.03628	1.91	0.04804
1389873_at	Pycard	PYD and CARD domain containing	1.44	0.01154	1.11	0.00233
1370215_at	C1qb	complement component 1, q subcomponent, B chain	1.44	0.01829	1.49	0.00008
1393347_at	Itgal	integrin, alpha L	1.42	0.03606	1.14	0.03066
1380537_at	Nckap1l	NCK associated protein 1 like	1.42	0.00055	1.18	0.00252
1367846_at	S100a4	s100 calcium-binding protein A4	1.41	0.02154	1.61	0.00468
1371482_at	LOC498279	similar to NADH dehydrogenase (ubiquinone) Fe-S protein 2	1.41	0.01444	1.47	0.00026
1380063_at	Ch25h	cholesterol 25-hydroxylase	1.39	0.04919	1.15	0.00995
1387627_at	Cd86	cd86 molecule	1.38	0.00576	1.46	0.01374
1368010_at	Ptpn6	protein tyrosine phosphatase, non-receptor type 6	1.38	0.02474	1.29	0.00171
1371079_at	Fcgr2b	Fc fragment of IgG, low affinity IIb, receptor (CD32)	1.36	0.01038	1.50	0.00334
1388267_a_at	Mt1a	metallothionein 1a	1.35	0.03417	1.88	0.00134
1368518_at	Cd53	cd53 molecule	1.34	0.01032	1.25	0.00003
1376652_at	C1qa	complement component 1, q subcomponent, A chain	1.33	0.02342	1.46	0.00411
1393219_at	C2	complement component 2	1.33	0.04749	1.40	0.03137
1374838_at	Sp140	SP140 nuclear body protein	1.32	0.00745	1.04	0.00892
1391637_at	LOC310190	rho GTPase activating protein 15	1.32	0.00837	1.21	0.01302
1377340_at	Tfpi2	tissue factor pathway inhibitor 2	1.32	0.01717	1.63	0.01599
1378180_at	LOC290741	dCMP deaminase	1.32	0.01100	0.84	0.02191
1382043_at	Unc93b1	unc-93 homolog B1 (C. elegans)	1.30	0.00961	0.92	0.01515
1384298_at	Myo1f	myosin IF	1.29	0.03092	1.32	0.00245
1369382_at	Mertk	c-mer proto-oncogene tyrosine kinase	1.29	0.02870	1.14	0.00534
1379760_at	Tnfaip8l2	tumor necrosis factor, alpha-induced protein 8-like 2	1.27	0.00014	0.89	0.02152
1373669_at	Gnpda2	glucosamine-6-phosphate deaminase 2	1.26	0.03160	1.78	0.00220
1388784_at	Csf1r	colony stimulating factor 1 receptor	1.26	0.00649	1.10	0.00837
1387198_at	Inpp5d	inositol polyphosphate-5-phosphatase D	1.26	0.00153	1.21	0.00709
1387195_at	St14	suppression of tumorigenicity 14 (colon carcinoma)	1.24	0.00075	1.46	0.00386
1369204_at	Hck	hemopoietic cell kinase	1.22	0.02925	0.86	0.00755
1369186_at	Casp1	caspase 1	1.20	0.00171	1.00	0.00374
1370885_at	Ctsz	cathepsin Z	1.20	0.03633	1.25	0.00414
1394134_at	LOC499337	dedicator of cytokinesis 8	1.20	0.02670	1.24	0.00388
1386987_at	Il6ra	interleukin 6 receptor, alpha	1.19	0.00440	0.88	0.03355
1395663_at	Mall	mal, T-cell differentiation protein-like	1.18	0.02080	1.37	0.00048
1370407_at	Pcyox1	prenylcysteine oxidase 1	1.18	0.02997	1.61	0.02707
1367974_at	Anxa3	annexin A3	1.15	0.00403	1.00	0.01502
1373204_at	Tmem176a	transmembrane protein 176A	1.15	0.00329	1.28	0.00018
1382531_at	Tlr7	toll-like receptor 7	1.14	0.02825	0.98	0.03055
1378633_at	Lpxn	leupaxin	1.13	0.03528	1.12	0.02954
1392648_at	Mrc2	mannose receptor, C type 2	1.13	0.04010	1.36	0.01450
1385324_x_at	RGD1561413	similar to BC021442 protein	1.12	0.01048	1.24	0.04768
1370219_at	Cyba	cytochrome b-245, alpha polypeptide	1.12	0.01176	1.11	0.00747
1367786_at	Psmb8	proteasome (prosome, macropain) subunit, beta type 8 (large multifunctional peptidase 7)	1.10	0.02084	0.83	0.02219
1386125_at	Hoxb4	homeo box B4	1.10	0.03464	1.00	0.01585
1388792_at	Gadd45g	growth arrest and DNA-damage-inducible, gamma	1.09	0.00358	0.75	0.02457
1380521_at	Hcls1	hematopoietic cell specific Lyn substrate 1	1.08	0.00519	1.17	0.00139
1382311_at	LOC310877	TRAF-interacting protein with forkhead-associated domain	1.07	0.04138	0.74	0.03675
1382680_at	Adfp	adipose differentiation related protein	1.07	0.01147	1.12	0.00913

Probe ID	Gene symbol	Gene full name	1W Log2 (fold change)	*p* Value	3W Log2 (fold change)	*p* Value
1390226_at	RGD1562552	similar to hypothetical protein LOC340061	1.07	0.01678	0.91	0.02630
1389885_at	Limd2	LIM domain containing 2	1.07	0.02417	0.78	0.03429
1372042_at	Cmtm3	CKLF-like MARVEL transmembrane domain containing 3	1.06	0.01772	1.02	0.00683
1390050_at	LOC682869	similar to Golgi phosphoprotein 2 (Golgi membrane protein GP73)	1.06	0.00531	0.90	0.00032
1391351_at	LOC680133	similar to TBC1 domain family, member 8	1.05	0.03702	1.04	0.04521
1383131_at	Itgb2	integrin, beta 2	1.03	0.00778	1.30	0.00864
1383780_at	Hectd2	HECT domain containing 1	1.03	0.00760	0.87	0.03970
1372013_at	Ifitm1	interferon induced transmembrane protein 1	1.01	0.02750	1.27	0.01786
1377950_at	RGD1309362	similar to interferon-inducible GTPase	1.01	0.03662	1.35	0.02164
1383658_at	Laptm5	lysosomal protein transmembrane 5	1.01	0.01825	1.09	0.00040
1388562_at	Stard7	StAR-related lipid transfer (START) domain containing 7	1.01	0.00022	0.84	0.00162
1385605_at	LOC688730	G protein-coupled receptor 84	1.00	0.00087	0.90	0.00130
1385605_at	LOC684132	similar to G protein-coupled receptor 84	1.00	0.00087	0.90	0.00130
1379957_at	Slfn8	schlafen 8	1.00	0.00660	0.66	0.02624
1389408_at	Rrm2	ribonucleotide reductase M2	0.99	0.03082	1.26	0.02806
1392834_at	Chd1l	chromodomain helicase DNA binding protein 1-like	0.99	0.02946	1.04	0.02032
1383011_at	Eif2a	eukaryotic translation initiation factor 2A	0.99	0.02964	1.15	0.02933
1368190_at	Ren	renin	0.98	0.02643	1.01	0.00349
1368840_at	Tmem176b	transmembrane protein 176B	0.98	0.00365	1.04	0.00125
1369611_at	Bcl2l2	bcl2-like 2	0.97	0.01814	0.95	0.00826
1373523_at	Fcgr3	Fc fragment of IgG, low affinity IIIa	0.96	0.00863	0.97	0.00159
1371583_at	Rbm3	RNA binding motif (RNP1, RRM) protein 3	0.96	0.00521	1.17	0.02956
1397907_at	LOC501736	Cd300a molecule	0.96	0.01279	1.17	0.00970
1373421_at	Tgif1	TGFB-induced factor homeobox 1	0.95	0.03898	1.08	0.04339
1383372_at	Ptafr	platelet-activating factor receptor	0.94	0.02968	0.91	0.00216
1390050_at	LOC680692	similar to Golgi phosphoprotein 2 (Golgi membrane protein GP73)	0.94	0.00657	0.74	0.00116
1370186_at	Psmb9	proteasome (prosome, macropain) subunit, beta type 9 (large multifunctional peptidase 2)	0.91	0.01743	0.74	0.02271
1383095_at	LOC681292	hypothetical protein LOC681292	0.91	0.00511	0.79	0.00372
1386899_at	Ctsh	cathepsin H	0.89	0.00328	0.94	0.00084
1386893_at	Grn	granulin	0.89	0.00776	1.18	0.00077
1368012_at	Tep1	telomerase associated protein 1	0.89	0.00472	0.84	0.00230
1374565_at	Nek6	NIMA (never in mitosis gene a)-related kinase 6	0.89	0.00511	0.79	0.02160
1390569_at	Cndp1	carnosine dipeptidase 1 (metallopeptidase M20 family)	0.85	0.00050	0.74	0.01381
1371256_at	Ptpn18	protein tyrosine phosphatase, non-receptor type 18	0.85	0.01833	0.65	0.03469
1388699_at	Man2b1	mannosidase, alpha, class 2B, member 1	0.84	0.01137	0.83	0.02209
1397399_at	Parp12	poly (ADP-ribose) polymerase family, member 12	0.84	0.02761	0.88	0.02132
1383407_at	Sash3	SAM and SH3 domain containing 3	0.84	0.03668	0.83	0.01183
1370449_at	P2ry13	purinergic receptor P2Y, G-protein coupled, 13	0.83	0.03023	0.66	0.00713
1375211_at	Rnaset2	ribonuclease T2	0.82	0.01711	0.81	0.00056
1385733_at	Exo1	exonuclease 1	0.82	0.02126	1.11	0.04599
1376776_at	LOC500528	coiled-coil domain containing 17	0.82	0.01478	0.86	0.02466
1371037_at	Pros1	protein S (alpha)	0.82	0.00840	0.81	0.00930
1382707_at	Rps6ka3	ribosomal protein S6 kinase polypeptide 3	0.81	0.00157	1.21	0.00001
1373443_a_at	Ankra2	ankyrin repeat, family A (RFXANK-like), 2	0.81	0.01608	1.19	0.01922
1376579_at	Lap3	leucine aminopeptidase 3	0.81	0.00645	1.38	0.01541
1386982_at	Mgat2	mannosyl (alpha-1,6-)-glycoprotein beta-1,2-N-acetylglucosaminyltransferase	0.80	0.02068	0.94	0.03473
1398453_at	LOC498957	kelch-like family member 36	0.80	0.00136	0.75	0.00142
1383248_at	Fmo5	flavin containing monooxygenase 5	0.80	0.04368	0.81	0.00145
1377899_at	RGD1304982	similar to RIKEN cDNA 2810025M15	0.79	0.02109	0.66	0.02428
1377369_at	Cybrd1	cytochrome b reductase 1	0.78	0.00252	1.04	0.02479
1370891_at	Cd48	cd48 molecule	0.78	0.01258	1.36	0.04950
1368332_at	Gbp2	guanylate binding protein 2	0.77	0.00042	0.88	0.00733
1370254_at	Clic6	chloride intracellular channel 5	0.76	0.03163	1.02	0.03416
1383673_at	Nap1l2	nucleosome assembly protein 1-like 2	0.76	0.04373	1.00	0.04708
1390707_at	Rgs10	regulator of G-protein signaling 10	0.76	0.01622	0.65	0.01951
1367515_at	Cnot7	CCR4-NOT transcription complex, subunit 7	0.75	0.03380	0.87	0.01289
1376795_at	Pik3ap1	phosphoinositide-3-kinase adaptor protein 1	0.74	0.03990	0.87	0.02528
1398892_at	Npc2	niemann-Pick disease, type C2	0.73	0.00542	0.88	0.00064
1392189_at	Rfx4	regulatory factor X, 4 (influences HLA class II expression)	0.73	0.03452	0.79	0.03114
1367679_at	HLA-DMB	major histocompatibility complex, class II, DM beta	0.73	0.02672	0.72	0.00755
1391355_at	LOC679389	hypothetical protein LOC679389	0.72	0.02751	0.86	0.04664
1393043_at	Utp18	UTP18, small subunit (SSU) processome component, homolog (yeast)	0.72	0.04853	0.96	0.03546
1387005_at	Ctss	cathepsin S	0.70	0.00157	0.76	0.00184
1394819_at	Sp110	SP110 nuclear body protein	0.70	0.03213	0.61	0.03686
1374061_at	Cd302	cd302 molecule	0.69	0.03300	0.74	0.04312
1375921_at	Rcn1	reticulocalbin 1, EF-hand calcium binding domain	0.69	0.02632	0.66	0.02794
1390472_at	Naaa	N-acylethanolamine acid amidase	0.69	0.00725	0.75	0.00904
1373252_at	LOC300225	similar to formin-like 3 protein	0.69	0.01206	0.66	0.00804
1389294_at	Cyfip1	cytoplasmic FMR1 interacting protein 1	0.67	0.00537	0.64	0.02893
1383241_at	C1r	complement component 1, r subcomponent	0.67	0.01359	0.96	0.01598
1372112_at	RGD1304579	tripartite motif-containing 5	0.65	0.00218	0.67	0.02462
1388455_at	Gng10	guanine nucleotide binding protein (G protein), gamma 10	0.64	0.04157	0.64	0.02737
1383089_at	Rab21	RAB21, member RAS oncogene family	0.64	0.02304	0.71	0.02777
1388140_at	Rab13	RAB13, member RAS oncogene family	0.63	0.02674	0.77	0.00251
1368728_at	P2ry12	purinergic receptor P2Y, G-protein coupled, 12	0.63	0.00089	0.68	0.01970
1389229_at	Acpl2	acid phosphatase-like 2	0.63	0.02464	0.66	0.04092
1367794_at	A2m	alpha-2-macroglobulin	0.62	0.01430	0.60	0.00152
1384017_at	Lrrk1	Leucine-rich repeat kinase 1	0.60	0.03486	0.61	0.00962
1373222_at	Hexa	hexosaminidase A	0.60	0.00791	0.76	0.00493
1375228_at	Brd2	bromodomain containing 2	0.60	0.00512	0.66	0.00972
1391463_at	Ddx58	DEAD (Asp-Glu-Ala-Asp) box polypeptide 58	0.59	0.00986	0.62	0.04171

Probe ID	Gene symbol	Gene full name	1W Log2 (fold change)	*p* Value	3W Log2 (fold change)	*p* Value
**Downregulated genes**						
1369715_at	Slc6a11	solute carrier family 6 (neurotransmitter transporter, GABA), member 11	-1.34	0.04452	-1.75	0.01381
1369667_at	Vps52	vacuolar protein sorting 52 homolog (S. cerevisiae)	-1.28	0.00935	-1.17	0.02778
1387155_at	Pcsk2	proprotein convertase subtilisin/kexin type 2	-1.23	0.04643	-1.74	0.01628
1391856_at	Sema3g	sema domain, immunoglobulin domain (Ig), short basic domain, secreted, (semaphorin) 3G	-1.12	0.02136	-1.06	0.02515
1369102_at	Mapk10	mitogen activated protein kinase 10	-1.03	0.00145	-0.91	0.01514
1369054_at	Rph3a	rabphilin 3A	-0.98	0.01792	-0.96	0.01126
1382067_at	LOC685859	UBX domain protein 2A	-0.98	0.04772	-1.11	0.04262
1381542_at	LOC679377	similar to UBX domain containing 4	-0.98	0.04772	-1.11	0.04262
1370901_at	RGD1306215	similar to hypothetical protein MGC36831	-0.96	0.01156	-0.82	0.02557
1384095_at	Myrip	myosin VIIA and Rab interacting protein	-0.95	0.04026	-1.04	0.04151
1390815_at	Cnnm1	Cyclin M1	-0.94	0.02603	-1.05	0.02732
1392749_at	Cntfr	ciliary neurotrophic factor receptor	-0.92	0.02624	-0.89	0.00726
1394668_at	Cadm3	cell adhesion molecule 3	-0.89	0.01867	-0.91	0.02290
1369046_at	Syt6	synaptotagmin VI	-0.88	0.00083	-0.82	0.00912
1376972_at	Slc40a1	solute carrier family 39 (iron-regulated transporter), member 1	-0.88	0.04083	-0.71	0.04196
1384652_at	Htatsf1	HIV-1 Tat specific factor 1	-0.88	0.00013	-0.84	0.03041
1397696_at	RGD1307830	similar to cDNA sequence BC003331	-0.86	0.00298	-0.97	0.03178
1389725_at	Tm7sf2	transmembrane 7 superfamily member 2	-0.85	0.00250	-0.71	0.00299
1382608_at	Actl6b	actin-like 6B	-0.85	0.02632	-1.11	0.01624
1393947_at	Slc25a15	solute carrier family 25 (mitochondrial carrier; ornithine transporter) member 15	-0.85	0.03586	-1.13	0.02234
1391748_at	P42pop	mypop Myb-related transcription factor, partner of profilin	-0.84	0.00044	-0.64	0.01563
1393353_at	RGD1304810	similar to 6430573F11Rik protein	-0.83	0.01621	-1.06	0.00769
1370125_at	Hapln1	hyaluronan and proteoglycan link protein 1	-0.81	0.01974	0.88	0.03019
1392882_at	RGD1305110	similar to KIAA1841 protein	-0.80	0.04792	-0.97	0.03848
1395008_at	Itsn1	intersectin 1 (SH3 domain protein)	-0.80	0.01961	-0.73	0.03721
1368572_a_at	Grin1	glutamate receptor, ionotropic, N-methyl D-aspartate 1	-0.77	0.02485	-0.89	0.02307
1380289_at	LOC691468	similar to Zinc finger protein 84 (Zinc finger protein HPF2)	-0.77	0.01453	-0.61	0.02927
1387020_at	Cyp51	cytochrome P450, family 51	-0.77	0.00328	-0.75	0.00344
1395985_at	Spg7	spastic paraplegia 7 homolog (human)	-0.76	0.00356	-0.74	0.02765
1385912_at	Dusp15	dual specificity phosphatase 15	-0.75	0.02872	-0.78	0.04789
1375188_at	Katnb1	katanin p80 (WD repeat containing) subunit B 1	-0.75	0.01289	-0.84	0.00136
1377920_at	Sgtb	small glutamine-rich tetratricopeptide repeat (TPR)-containing, beta	-0.75	0.00737	-0.98	0.01477
1387068_at	Arc	activity-regulated cytoskeleton-associated protein	-0.74	0.00260	-0.81	0.00217
1374608_at	LOC500295	DNase domain containing 2	-0.74	0.04158	-0.63	0.00913
1385069_at	Acvr1	activin A receptor, type I	-0.74	0.00274	-0.95	0.00374
1369781_at	Grm7	glutamate receptor, metabotropic 7	-0.73	0.02614	-0.97	0.01380
1372617_at	Btbd2	BTB (POZ) domain containing 2	-0.71	0.02613	-0.78	0.00323
1372841_at	Reep6	receptor accessory protein 6	-0.70	0.04454	-0.85	0.02030
1389634_at	Metap1	methionyl aminopeptidase 1	-0.69	0.04329	-1.07	0.02714
1369180_at	Bcl2l1	Bcl2-like 1	-0.69	0.01611	-0.84	0.00799
1387976_at	Slc9a3r2	solute carrier family 9 (sodium/hydrogen exchanger), member 3 regulator 2	-0.68	0.00415	-0.76	0.02156
1390513_at	LOC687609	similar to ras homolog gene family, member f	-0.67	0.00187	-0.71	0.01039
1397861_at	Vstm2b	V-set and transmembrane domain containing 2B	-0.65	0.01260	-0.69	0.00850
1392967_at	MGC116121	similar to RIKEN cDNA 2700062C07	-0.64	0.03161	-0.62	0.03544
1387676_at	Mark1	MAP/microtubule affinity-regulating kinase 1	-0.63	0.01438	-1.01	0.00996
1370144_at	LOC689842	similar to Nucleolar GTP-binding protein 1 (Chronic renal failure gene protein) (GTP-binding prote	-0.63	0.02628	-0.72	0.01606
1369813_at	Dnajc5	dnaJ (Hsp40) homolog, subfamily C, member 5	-0.61	0.04069	-1.07	0.01613
1368425_at	Caskin1	CASK interacting protein 1	-0.61	0.03034	-0.65	0.01883
1384741_at	Pla2g3	phospholipase A2, group III	-0.61	0.00838	-0.64	0.01325
1396409_at	Dtnb	dystrobrevin, beta	-0.61	0.01074	-0.71	0.01782
1385526_at	LOC365601	autophagy related 5	-0.60	0.00953	-0.75	0.03545
1370208_at	Nr1h2	nuclear receptor subfamily 1, group H, member 2	-0.60	0.03252	-0.66	0.02612
1375401_at	Mvk	mevalonate kinase	-0.60	0.00057	-0.67	0.00058
1388536_at	Bat2	HLA-B associated transcript 2	-0.60	0.00069	-0.75	0.02364
1369086_a_at	Cacna1d	calcium channel, voltage-dependent, L type, alpha 1D subunit	-0.60	0.04068	-0.74	0.02200
1375580_at	Dpp10	dipeptidyl peptidase 10	-0.59	0.02525	-0.72	0.00746
1381406_at	RGD1561734	similar to KIAA1913	-0.59	0.04027	-0.81	0.01109
