# Supplementary material for: Molecular and Cellular Changes in the Lumbar Spinal Cord following Thoracic Injury: Regulation by Treadmill Locomotor Training
Source: PLoS One. 2014 Feb 10;9(2):e88215. doi: 10.1371/journal.pone.0088215 (PMC3919755; doi:10.1371/journal.pone.0088215)
Supplement: Table S4 — A list of genes that were differentially regulated by treadmill training in sham- operated animals. (PDF) [file pone.0088215.s004.pdf]

Table S4. A list of genes that were differentially regulated by treadmill training in sham operated animals

Probe ID	Gene symbol	Gene full name	Log2 (fold change)	*p* Value
**Upregulated genes**				
1387828_at	Agap2	ArfGAP with GTPase domain, ankyrin repeat and PH domain 2	2.72	0.02335
1373195_at	Fus	fusion (involved in t(12;16) in malignant liposarcoma) (human)	1.37	0.01480
1391815_at	Rpusd3	RNA pseudouridylate synthase domain containing 3	1.31	0.01334
1398388_at	UNK	unkempt homolog (Drosophila)	1.28	0.02185
1373409_at	UBE3C	ubiquitin protein ligase E3C	1.11	0.00132
1395715_at	Cdgap	cdc42 GTPase-activating protein	1.06	0.00948
1367771_at	Tsc22d3	TSC22 domain family, member 3	1.02	0.00356
1383647_a_at	Crb3	crumbs homolog 3 (Drosophila)	1.01	0.02292
1392648_at	MRC2	mannose receptor, C type 2	1.01	0.04829
1371092_at	LOC286991	putative retrovirus-related gag protein	1.00	0.04291
1388297_at	Eef1g	eukaryotic translation elongation factor 1 gamma	0.98	0.00036
1367589_at	Aco2	aconitase 2, mitochondrial	0.95	0.00003
1378914_a_at	LOC308990	hypothetical protein LOC308990	0.95	0.00870
1387762_s_at	Jund	jun D proto-oncogene	0.91	0.04944
1389271_at	LOC679921	vasorin	0.90	0.03177
1391019_at	LOC687214	similar to Slit-like 2	0.90	0.03177
1375637_at	RGD1311122	similar to RIKEN cDNA 1110003E01	0.88	0.00102
1399070_at	LOC691036	similar to SET binding factor 2	0.86	0.01853
1370045_at	Polg	polymerase (DNA directed), gamma	0.86	0.00787
1368171_at	Lox	lysyl oxidase	0.85	0.01468
1376407_a_at	Lsm7	LSM7 homolog, U6 small nuclear RNA associated (S. cerevisiae)	0.85	0.02066
1398843_at	Vapa	VAMP (vesicle-associated membrane protein)-associated protein A	0.85	0.00252
1385062_at	RGD1306072	hypothetical LOC304654	0.84	0.04598
1395737_at	Cd276	cd276 molecule	0.84	0.01144
1393510_at	Golsyn	golgi-localized protein	0.82	0.00055
1380517_at	Zmym3	zinc finger, MYM-type 3	0.82	0.01201
1395735_at	RGD1310271	similar to hypothetical protein MGC45873	0.82	0.02404
1373598_at	UBN1	ubinuclein 1	0.81	0.00181
1390569_at	Cndp1	carnosine dipeptidase 1 (metallopeptidase M20 family)	0.77	0.03893
1398453_at	LOC498957	kelch-like family member 36	0.77	0.00891
1389469_at	Chd1l	chromodomain helicase DNA binding protein 1-like	0.75	0.00710
1389095_at	Boc	biregional cell adhesion molecule-related/down-regulated by oncogenes (Cdon) binding protein	0.73	0.03763
1377967_at	Cdt1	chromatin licensing and DNA replication factor 1	0.72	0.04509
1386160_at	Tchh	trichohyalin	0.72	0.03102
1374478_at	RGD1305347	similar to RIKEN cDNA 2610528J11	0.71	0.04133
1371074_a_at	Mcm6	minichromosome maintenance complex component 6	0.70	0.00027
1374456_at	LOC682900	hypothetical protein LOC682900	0.70	0.04117
1368279_at	LOC679252	similar to Myeloid/lymphoid or mixed-lineage leukemia protein 3 homolog (Histone-lysine N-methyltransferase, H3 lysine-4 specific MLL3)	0.68	0.00197
1370908_at	HDAC7A	histone deacetylase 7	0.68	0.04534
1372312_at	Ltv1	LTV1 homolog (S. cerevisiae)	0.68	0.02653
1376259_at	Prkcq	protein kinase C, theta	0.68	0.03199
1387195_at	St14	suppression of tumorigenicity 14 (colon carcinoma)	0.67	0.01803
1368187_at	Gpnmb	glycoprotein (transmembrane) nmb	0.67	0.00511
1386987_at	Il6ra	interleukin 6 receptor, alpha	0.67	0.01844
1387874_at	Dbp	D site of albumin promoter (albumin D-box) binding protein	0.66	0.00494
1368717_at	Faah	fatty acid amide hydrolase	0.66	0.01965
1369161_at	Abcb4	ATP-binding cassette, sub-family B (MDR/TAP), member 4	0.66	0.03459
1393095_at	Tnks1bp1	tankyrase 1 binding protein 1	0.64	0.04910
1398506_at	Ppp3ca	Protein phosphatase 3 (formerly 2B), catalytic subunit, alpha isoform	0.63	0.03422
1385282_at	Nat14	N-acetyltransferase 14	0.62	0.02947
1386938_at	Anpep	alanyl (membrane) aminopeptidase	0.61	0.04808
1383017_at	Ptprm	protein tyrosine phosphatase, receptor type, M	0.61	0.00527
1374025_at	Nmnat3	nicotinamide nucleotide adenylyltransferase 3	0.61	0.00868
1391637_at	LOC295635	Rho GTPase activating protein 15	0.61	0.02610
1369614_at	Rap2b	RAP2B, member of RAS oncogene family	0.61	0.01152
1376977_at	Ptger3	Prostaglandin E receptor 3	0.60	0.02624
1384103_at	RGD1561416	similar to novel protein (HT036)	0.60	0.03134
1387718_at	P2rx7	purinergic receptor P2X, ligand-gated ion channel, 7	0.60	0.00421
1378140_at	Arl11	ADP-ribosylation factor-like 11	0.60	0.03299
1387247_at	Pcsk1	proprotein convertase subtilisin/kexin type 1	0.59	0.03824
**Downregulated genes**				
1375362_at	Sppl2a	signal peptide peptidase-like 2A	-3.00	0.00771
1370545_at	Kcna1	potassium voltage-gated channel, shaker-related subfamily, member 1	-2.61	0.00733
1376654_at	RGD1308448	similar to RIKEN cDNA B130016O10 gene	-2.51	0.01592
1373046_at	Boll	bol, boule-like (Drosophila)	-2.38	0.00185
1368902_at	Pak3	p21 protein (Cdc42/Rac)-activated kinase 3	-1.95	0.01593
1391075_at	Rgs17	regulator of G-protein signaling 17	-1.82	0.03396
1395326_at	Rbm9	RNA binding motif protein 9	-1.76	0.00908
1368841_at	Tcf4	transcription factor 4	-1.75	0.01095
1378740_at	Rasal2	RAS protein activator like 2	-1.74	0.01789
1375259_at	Eif4ebp2	eukaryotic translation initiation factor 4E binding protein 2	-1.73	0.00063
1370520_at	LOC257650	hippyragranin	-1.73	0.02396
1375280_at	Fam152a	desumoylating isopeptidase 2	-1.68	0.00520
1383829_at	Bbx	bobby sox homolog (Drosophila)	-1.63	0.01513
1376436_at	Lysmd3	putative peptidoglycan-binding, domain containing 3	-1.56	0.01700
1376523_at	Arid4a	AT rich interactive domain 4A (Rbp1 like)	-1.55	0.02867
1377926_at	Agap1	ArfGAP with GTPase domain, ankyrin repeat and PH domain 1	-1.50	0.02466
1391347_at	Rab8b	RAB8B, member RAS oncogene family	-1.48	0.01429
1392453_at	Clcn3	chloride channel 3	-1.45	0.02845
1396131_at	Fam134c	family with sequence similarity 134, member C	-1.41	0.00966
1387204_at	Negr1	neuronal growth regulator 1	-1.38	0.02257
1377061_at	Rics	rho GTPase-activating protein	-1.38	0.04416
1389978_at	LOC500378	chromatin target of PRMT1	-1.33	0.01436
1368958_at	Pacsin1	protein kinase C and casein kinase substrate in neurons 1	-1.33	0.03412
1394042_at	LOC682864	similar to 82-kD FMRP Interacting Protein	-1.19	0.00183
1391405_at	Thsd7a	thrombospondin, type I, domain containing 7A	-1.16	0.04559
1390097_at	Tspyl4	TSPY-like 4	-1.15	0.00144
1369715_at	Slc6a11	solute carrier family 6 (neurotransmitter transporter, GABA), member 11	-1.13	0.04524
1376582_at	Med1	mediator complex subunit 1	-1.12	0.01483
1376644_at	Med19	mediator complex subunit 19	-1.12	0.02533
1383296_a_at	Xpo4	exportin 4	-1.09	0.00357
1394714_at	Atp2b1	ATPase, Ca++ transporting, plasma membrane 1	-1.08	0.02325
1383112_at	Trps1	trichorhinophalangeal syndrome I homolog (human)	-1.08	0.04632

Probe ID	Gene symbol	Gene full name	Log2 (fold change)	*p* Value
1387024_at	Dusp6	dual specificity phosphatase 6	-1.07	0.00018
1390179_at	Ankrd52	ankyrin repeat domain 52	-1.07	0.04940
1393910_at	Fam13a1	family with sequence similarity 13, member A1	-1.06	0.03831
1376921_at	Foxred1	FAD-dependent oxidoreductase domain containing 1	-1.05	0.02386
1369304_at	Pts	6-pyruvoyl-tetrahydropterin synthase	-1.05	0.04936
1370016_at	Nell2	NEL-like 2 (chicken)	-1.02	0.00129
1377042_at	LOC681178	polycomb group ring finger 5	-1.00	0.03551
1373177_x_at	LOC501485	hypothetical LOC501485	-1.00	0.04615
1369404_a_at	Nrxn1	neurexin 1	-0.99	0.01488
1370577_at	Zfp455	zinc finger protein 455	-0.96	0.03731
1373542_at	Sphk2	sphingosine kinase 2	-0.95	0.00193
1387807_at	Pafah1b1	platelet-activating factor acetylhydrolase, isoform 1b, subunit 1	-0.95	0.01540
1388646_at	Depdc6	DEP domain containing MTOR-interacting protein	-0.94	0.00312
1390099_at	Ube3a	ubiquitin protein ligase E3A	-0.93	0.02336
1388885_at	Aph1a	anterior pharynx defective 1 homolog A (C. elegans)	-0.90	0.04092
1377325_a_at	LOC685076	phosphoglucomutase 2-like 1	-0.90	0.02886
1370008_at	Psmc3ip	PSMC3 interacting protein	-0.88	0.04213
1392472_at	LOC309957	myocyte enhancer factor 2a	-0.87	0.02467
1370160_at	Xpnpep1	X-prolyl aminopeptidase (aminopeptidase P) 1, soluble	-0.86	0.00377
1385692_at	Slc10a4	solute carrier family 10 (sodium/bile acid cotransporter family), member 4	-0.86	0.00097
1374968_at	Eif2c1	eukaryotic translation initiation factor 2C, 1	-0.85	0.00146
1384797_at	Atl3	atlastin GTPase 3	-0.84	0.03538
1395074_at	Tdg	thymine-DNA glycosylase	-0.83	0.04127
1387060_at	Klf6	Kruppel-like factor 6	-0.83	0.03239
1375785_at	Fktn	fukutin	-0.83	0.00576
1377713_at	Foxn3	forkhead box N3	-0.81	0.02659
1369248_a_at	Xiap	X-linked inhibitor of apoptosis	-0.81	0.02896
1370912_at	Hspa1a	heat shock 70kD protein 1A	-0.80	0.00671
1367996_a_at	Lphn1	latrophilin 1	-0.79	0.04174
1381887_x_at	RGD1311595	similar to KIAA2026 protein	-0.79	0.01104
1373497_at	Hipk2	homeodomain interacting protein kinase 2	-0.79	0.01677
1397439_at	LOC497978	similar to diacylglycerol kinase epsilon	-0.78	0.04424
1381462_at	RGD1566130	similar to mKIAA1940 protein	-0.77	0.04588
1382171_at	LOC499624	TSC22 domain family, member 2	-0.77	0.04328
1384516_at	Mtf2	metal response element binding transcription factor 2	-0.75	0.03509
1396096_at	Mgat4a	mannosyl (alpha-1,3-)-glycoprotein beta-1,4-N-acetylglucosaminyltransferase, isozyme A	-0.73	0.00294
1399070_at	Setd5	SET domain containing 5	-0.72	0.00418
1367952_at	Lrp2	low density lipoprotein-related protein 2	-0.69	0.04911
1375682_at	Pura	purine rich element binding protein A	-0.68	0.02906
1368903_at	Strbp	spermatid perinuclear RNA binding protein	-0.67	0.02367
1390055_at	RGD1306314	predicted similar to KIAA0819 protein (predicted)	-0.66	0.03016
1377582_at	Tox3	TOX high mobility group box family member 3	-0.66	0.03296
1376576_at	Dusp11	dual specificity phosphatase 11 (RNA/RNP complex 1-interacting)	-0.65	0.03091
1369491_at	Dao	D-amino-acid oxidase	-0.65	0.04924
1398588_at	LOC303057	SLU7 splicing factor homolog (S. cerevisiae)	-0.64	0.00310
1375575_at	Camk2b	calcium/calmodulin-dependent protein kinase II beta	-0.63	0.04114
1379218_at	LOC365949	similar to nemo like kinase	-0.63	0.02475
1390857_at	Xylb	xylulokinase homolog (H. influenzae)	-0.62	0.01318
1392560_at	Pip5k1a	phosphatidylinositol-4-phosphate 5-kinase, type 1, alpha	-0.60	0.00682
1376871_at	RGD1562140	similar to brain Zn-finger protein	-0.60	0.03539
1382265_at	Gcap14	granule cell antiserum positive 14	-0.59	0.03161
1387663_at	Gmfb	glia maturation factor, beta	-0.59	0.03126
